# Luminescent Iridium Complexes Used in Light-Emitting Electrochemical Cells (LEECs)

**DOI:** 10.1007/s41061-016-0036-0

**Published:** 2016-06-06

**Authors:** Adam F. Henwood, Eli Zysman-Colman

**Affiliations:** Organic Semiconductor Centre, EaStCHEM School of Chemistry, University of St Andrews, St Andrews, Fife, KY16 9ST UK

**Keywords:** Light-emitting electrochemical cells, Phosphorescence, Iridium, Electroluminescence

## Abstract

Cationic iridium(III) complexes represent the single largest class of emitters used in light emitting electrochemical cells (LEECs). In this chapter, we highlight the state-of-the-art emitters in terms of efficiency and stability in LEEC devices, highlighting blue, green, yellow/orange, red and white devices, and provide an outlook to the future of LEECs.

## Introduction

Luminescent materials based on iridium(III) complexes have become the “go to” material when designing emitters for solid-state lighting (SSL) applications. Since the first report of a phosphorescent emitter in an organic light-emitting diode (OLED) [1], iridium complexes have come to be the most widely used class of emitters employed for this purpose by virtue of their efficient spin–orbit coupling (SOC) processes that relax the spin selection rule that otherwise forbids T_1_ to S_0_ transitions [2–4]. In an electroluminescent device, spin statistics necessitates that 75 % of excitons generated in the device are in the triplet state, while the remainder are in the singlet state. Therefore, iridium complexes are able to harvest 100 % of the excitons.

Aside from OLEDs, an alternative class of organic lighting device that is gaining attention is the light-emitting electrochemical cell (LEEC) [5–8]. In contrast to OLEDs, where the emitter is typically a charge-neutral chromophore, LEECs based on phosphorescent emitters utilize intrinsically charged ionic transition metal complexes (iTMCs) as the emitters in the device. The charged nature of the emitters confers a unique operating mechanism to the LEEC, whereby application of a bias leads to a slow migration of the ions in the device to the relevant electrodes. As this migration occurs, the barrier to charge injection drops significantly, meaning that only low work-function electrodes such as Au, Ag, or Al are required to operate these devices. Using air-stable electrodes thereby enables these devices to be solution processed and thus make them potential candidates for industrial scale processing. However, despite the promise of solution processing, to date the performance metrics of LEECs (device efficiency, stability) have remained some way short of their OLED counterparts, and significant improvements are needed if these devices are to become widely adopted in the future.

In this chapter, we will first outline the use of the most frequently employed iridium complex in LEECs, [Ir(ppy)_2_(bpy)](PF_6_), **1**, (where ppyH is 2-phenylpyridine and bpy is 2,2′-bipyridine) and demonstrate why its photophysical properties make it an attractive candidate for LEEC applications. We will next detail all the examples where this emitter has been reported in a device and how the performance of different devices changes as a function of study design and device architecture. We will then use the well-understood properties of this complex as a reference to contrast the performance of LEECs employing new emitters that differ from **1** through modulation of the substituents on the ligand scaffolds. In doing so, we will identify champion devices, in terms of efficiency and stability, categorized according to the emission color of the device, covering: blue, green, yellow/orange, red and white devices. We will provide insight into how the physical and chemical properties of the emitters employed in these devices confer such good performances, with a view to informing future molecular design of emitters for LEECs. Finally, we will conclude by offering a perspective on LEECs for the future.

## [Ir(ppy)_2_(bpy)](PF_6_)

### Syntheses

**Fig. 1:**
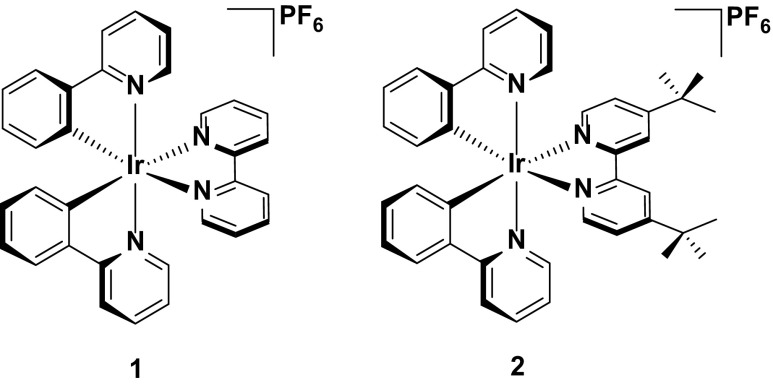
Structures of [Ir(ppy)_2_(bpy)](PF_6_), **1**, and its *tert*-butyl analogue, [Ir(ppy)_2_(dtbubpy)](PF_6_), **2**, which are widely studied iridium complexes employed in LEEC devices

One of the most extensively explored emitters for LEECs is the archetypal cationic iridium complex **1**. Although not the first example of an iridium complex tested in a LEEC, (this distinction goes to its cousin, [Ir(ppy)_2_(dtbubpy)](PF_6_), **2**, where dtbubpy is 4,4′-di-*tert*-butyl-2,2′-bipyridine) [9], the simple structure within this large family of cationic complexes makes it a useful reference to compare the performance of LEECs (Fig. 1).

**Scheme 1:**
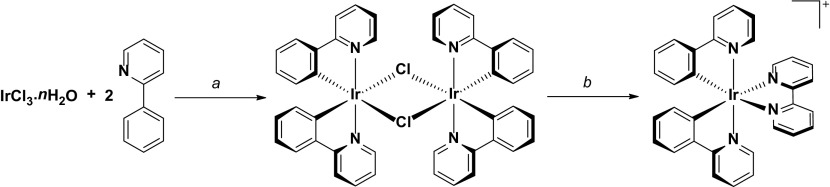
General protocol for the synthesis of [Ir(ppy)_2_(bpy)]^+^. ^a^2-EtO-C_2_H_4_OH/H_2_O (4:1 v/v), 110 °C, N_2_, 19 h. ^b^2,2′-bipyridyine (2.1 equiv.), CH_2_Cl_2_/MeOH (1:1 v/v), 40 °C, N_2_, 19 h

By far the most popular protocol for synthesizing this and related complexes is by the route shown in Scheme 1. The synthesis proceeds first by isolation of the μ-dichloro-bridged cyclometalated iridium dimer intermediate, following refluxing an iridium(III) salt in high boiling alcoholic solvents such as 2-ethoxyethanol, as first demonstrated by Nonoyama in 1974 [10]. Although Nonoyama demonstrated this synthesis using Na_3_[IrCl_6_] as the iridium source, IrCl_3_.*n*H_2_O is now by far the most popular source of iridium used for synthesizing these complexes. The dimer can then be easily cleaved in the presence of a neutral N^N ligand under mild conditions (such as refluxing DCM/methanol) [11], with the complex isolated as its chloride salt. While the chloride salts of these complexes do function LEECs, chloride anions have been implicated to negatively impact device stability (vide infra) [12] and thus typically the chloride anion is exchanged through a metathesis reaction for a tetrafluoroborate (BF_4_^−^) or most commonly a hexafluorophosphate (PF_6_^−^) anion instead.

**Scheme 2:**
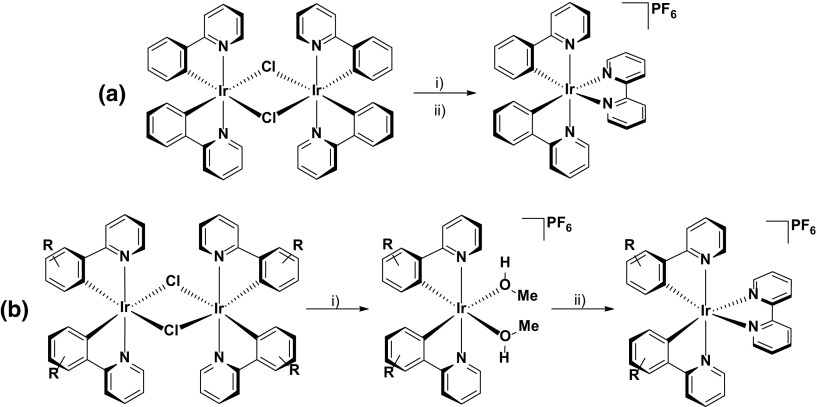
Two different protocols for isolating chloride-free [Ir(ppy)_2_(bpy)](PF_6_)-type complexes reported by Housecroft et al. [14]. **a** *(i)* 2,2’-bipyridine (2.0 equiv.), MeOH, MW 120 °C, 14 bar, 2 h; *(ii)* excess NH_4_PF_6_ and AgPF_6_, r.t., 1 h. **b** R is a 4-phenyl substituent or 4,6-diphenyl substituent *(i)* MeOH, AgPF_6_, r.t., 2 h; *(ii)* 2,2′-bipyridine (1.0 equiv.) MeOH, NH_4_PF_6_, r.t., 1 h

The performance of the LEEC can be severely impaired by the presence of trace impurities, such as chloride [12] or water [13]. Therefore, several groups have explored alternative synthetic protocols in order to minimize their presence. For example, the Housecroft group initially demonstrated that chloride-free [Ir(ppy)_2_(bpy)](PF_6_) could be isolated by performing the anion metathesis reaction with a mixture of NH_4_PF_6_ and AgPF_6_ [12]. Removal of AgCl by filtration over Celite is facile, leaving the PF_6_^−^ anion as the only counterion in the sample as confirmed by elemental analysis. Device studies verified the merits of this synthetic route, with the chloride-free samples showing luminance levels roughly double that of samples containing just 1 % chloride. The group’s synthetic methodology has evolved since this initial report, with recent efforts demonstrating that cleaving the μ-dichloro-bridged dimer with a silver salt in the presence of a weakly coordinating solvent allows for isolation of chloride-free solvento-complex intermediates, which can be further reacted with the ancillary ligand to afford the final complexes in excellent purity (and crucially, free of chloride counterions). Devices fabricated from complexes synthesized using this methodology showed exceptionally long-lived stability, with reported *t*_1/2_ values (time taken for the luminance to drop to half its maximum) in excess of 2800 h, even when operated at a notably high pulsed current density of 300 A m^−2^ [14]. These devices will be discussed in more detail in Sect. 5 (Scheme 2).

**Scheme 3:**
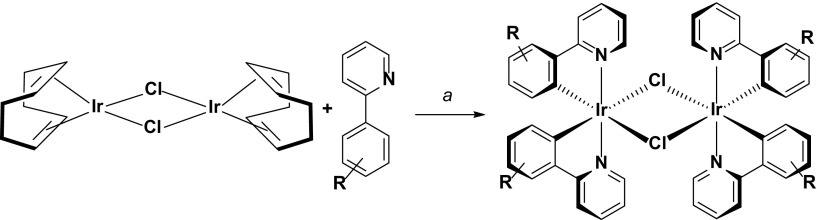
Protocol for synthesizing [Ir(ppy)_2_(μ-Cl)]_2_-type dimer complexes from [Ir(COD)(μ-Cl)]. R is either an unsubstituted ring or a 2,4-difluorophenyl substituted ring. ^a^2-EtO-C_2_H_4_OH, 110 °C, N_2_, 3 h

Alternatively, the Baranoff group has explored using iridium(I) precursor materials in lieu of the more conventional iridium(III) chloride salts. Often, iridium dimers isolated from the IrCl_3_.*n*H_2_O reaction are not pure, but are nevertheless used without purification in the subsequent cleavage step. Purification of the final complex usually involves chromatography and/or recrystallization. However, purifying in this manner can be arduous or sometimes not possible at all. The Baranoff group has shown that the dimer [Ir(COD)(μ-Cl)]_2_, bearing labile 1,4-cyclooctadiene (COD) ligands, is more amenable to cyclometalation, with reactions proceeding in shorter times (usually 3 h) and affording much cleaner isolated dimers that facilitate the final purification process [15, 16]. They have also reported improved device performance based on materials synthesized in this manner [17] (Scheme 3).

### Photophysics

**Fig. 2:**
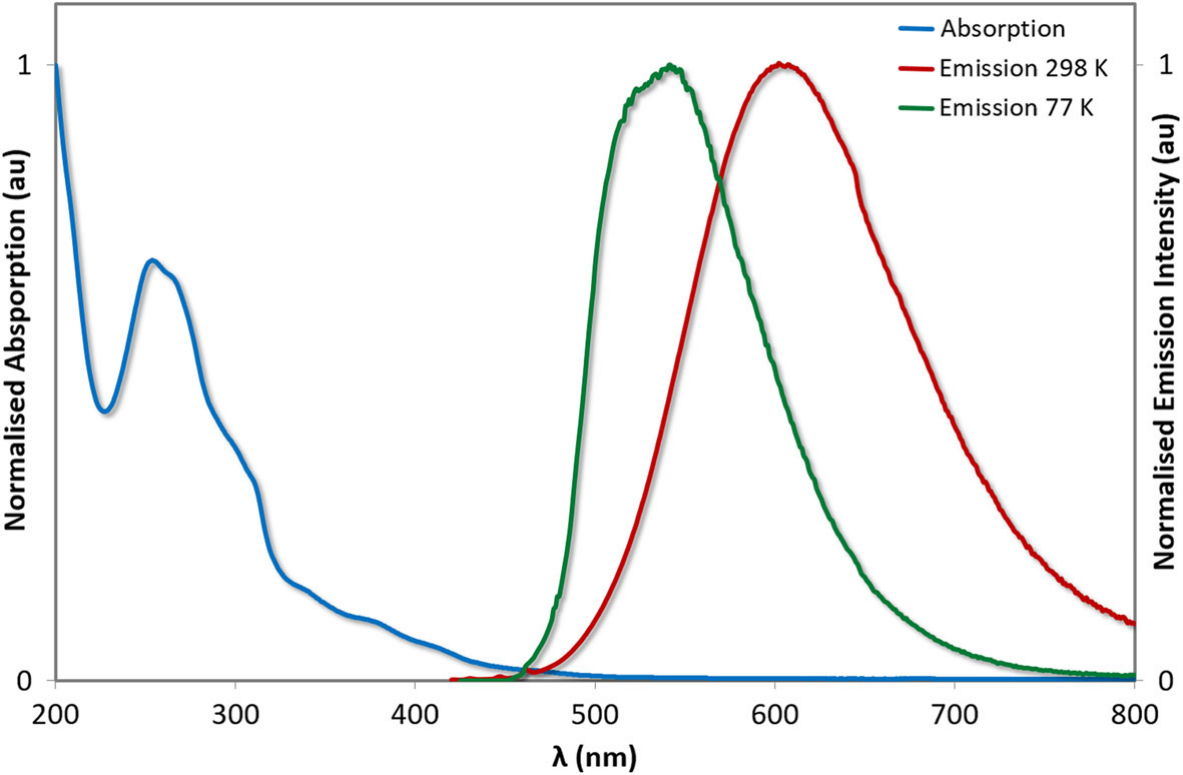
UV-Vis absorption spectra and emission spectra of **1** in MeCN. 77 K emission spectrum of **1** in 2-MeTHF

**Fig. 3:**
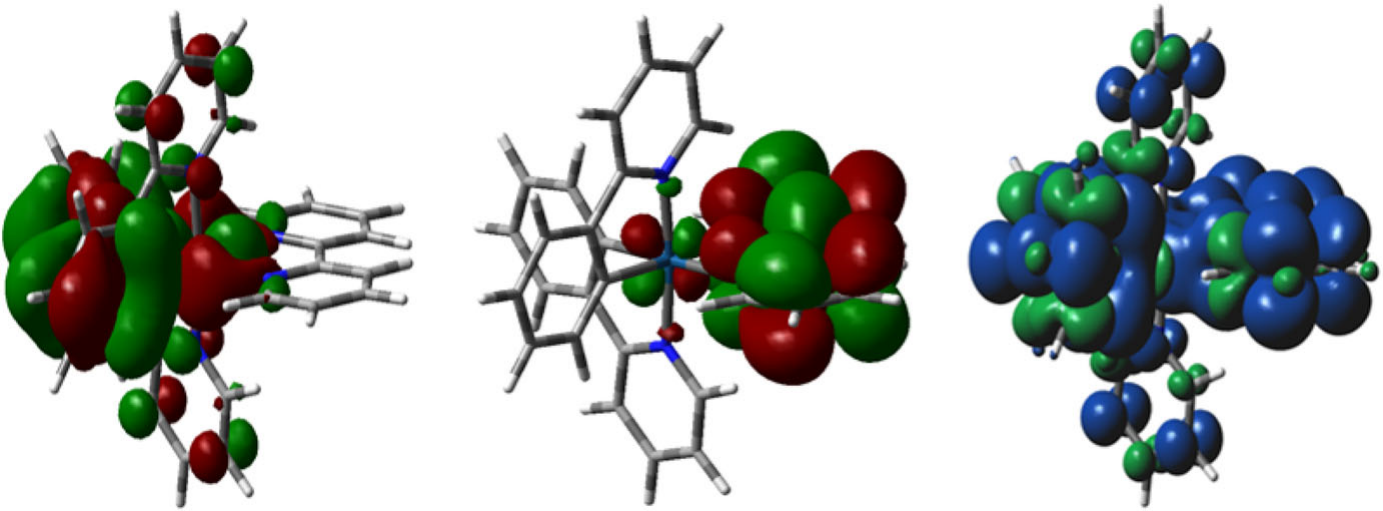
DFT computed Kohn–Sham orbitals for the HOMO (*left*) and LUMO (*middle*) of **1**. Spin density of the *T*_1_ state of **1** (*right*)

The relevant photophysical and electrochemical data for **1** is summarized in Table 1. In acetonitrile solution at room temperature **1** is an orange-yellow emitter with a broad, unstructured emission centered at 585 nm, with a triplet lifetime of 0.43 μs. This emission profile is characteristic of many [Ir(C^N)_2_(N^N)]^+^ complexes, identified by Güdel as comprising a mixed charge transfer (CT, Fig. 2) triplet excited state consisting of CT transitions between the metal and the N^N ancillary ligand (metal-to-ligand charge transfer, ^3^MLCT) and between the phenyl groups of the C^N ligands and the N^N ancillary ligand (ligand-to-ligand charge transfer, ^3^LLCT) [18–20]. The spin density of the triplet state is thus delocalized over the entire complex (Fig. 3). Upon cooling to 77 K, the emission is hypsochromically shifted but remains unstructured. This rigidochromic blue-shifting of the emission upon cooling is a further hallmark of the mixed CT nature of the emission, which is stabilized at ambient temperature by polar aprotic solvents such as MeCN. Aside from ^3^MLCT/^3^LLCT excited states, [Ir(C^N)_2_(N^N)]^+^ complexes can also demonstrate structured emission profiles that are attributed to centered radiative decay (^3^LC). It is not uncommon for cationic iridium complexes to exhibit emission from a mixture of ^3^LC and ^3^MLCT/^3^LLCT states [3].

**Table 1:** Relevant photophysical parameters for [Ir(ppy)_2_(bpy)](PF_6_), **1**

	**1**	References
*λ*_abs_/nm [ε (×10^4^/M^−1^ cm^−1^)]^a^	265 [4.17], 310 [1.29], 375 [0.60], 420 [0.26]	[27]
*λ*_em(sol)_/nm^a,b^	605	[22]
*λ*_em(film)_/nm^c^	587	[21]
*Φ*_PL(sol)_/%^b,d^	9	[22]
*Φ*_PL(film)_/%^c,e^	34	[21]
*Φ*_PL(film)_/%^e^	66	[23]
*τ*_e_/μs^a,b^	0.43	[21]

^a^Measured in MeCN at 298 K

^b^Measured under deaerated conditions

^c^Film composition: 1:1 iridium complex to ionic liquid

^d^Using Ru(bpy)_3_(PF_6_)_2_ as the standard (*Φ*_PL_ = 9.5 % in MeCN), and scaled according to this value

^e^Measured using an integrating sphere

The photophysical properties of many iridium complexes in the solid state are very different to the solution state. In the case of **1**, the emission energy in thin films compared to MeCN solution is virtually unchanged (a somewhat rare phenomenon), but the Φ_PL_ values differ dramatically. Reports of the Φ_PL_ of **1** in MeCN have been somewhat variable ranging from 6 to 14 % [21, 22], but ultimately are rather low, while in the ‘LEEC’ film (containing the complex and an ionic liquid, IL, additive in a 1:1 molar ratio) or in a doped film (5 wt % in PMMA) the Φ_PL_ values are substantially higher (34 % [21] and 66 % [23], respectively). The increased brightness in the solid state is plausibly attributed to rigidification of the local environment that inhibits molecular motions that otherwise non-radiatively deactivate the excited state. Although clearly a desirable feature, such effects are difficult to predict, with this rigidification phenomenon often in competition with self-quenching processes that lower the Φ_PL_. Self-quenching is particularly problematic for LEECs since, unlike OLEDs, the emitters are not normally doped into host matrices; typically, the emissive layers are neat films or contain small amounts of IL doped into the host emitter (4:1 and 1:1 weight by weight ratios are the most common configurations), leading to films that show lower Φ_PL_ compared to solution and thus lower device efficiencies as well. Furthermore, the emission energies of these complexes can change substantially in the solid state. Small red shifts frequently occur due to effects such as aggregate formation but on occasion substantial red-shifting (as high as 71 nm, or 2037 cm^−1^) [24] or even blue-shifting have been reported [25, 26]. The example given here for **1** demonstrates some of these effects, but they will be revisited in multiple instances throughout this chapter.

Aside from characterizing its photophysical parameters, it is important to determine the electrochemical properties of these complexes as well. Normally, this is done in order to estimate the energies of the HOMO and LUMO levels [28, 29]. These values are particularly important in the context of OLEDs so that the energy levels of the emissive material can properly align with those of the host materials and charge transport layers. For LEECs, this consideration is only important when aligning the energy levels of host–guest systems [30–32], but not for more traditional ‘single emitter’ devices since the emitters here also carry out the role of charge transport. The dual charge transport/light-emitting role of the iTMCs in the device is the most important contributing factor for explaining why LEECs are invariably less stable than their OLED counterparts. Thus, an important feature to look for when characterizing the electrochemical properties of the emitter is for reversible oxidation and reduction waves since good reversibility suggests that the emitter might be more resilient to electrochemical degradation when operating in the device.

With the support of DFT calculations (Fig. 3), the nature of the oxidation and reduction of **1** has been assigned. The oxidation is ascribed to the Ir^III/IV^ redox couple combined with contribution from the phenyl rings of the cyclometalating ligands. The degree of reversibility of this redox couple depends on the magnitude of the contribution from the C^N ligands; greater contributions results in a greater degree of irreversible electrochemistry. The reduction is assigned to a highly reversible bpy^0/1−^ redox couple, which is believed to be an important factor in giving devices based on **1** impressive stability metrics (*t*_1/2_ = 668 h, vide infra) [23].

### Devices

To the best of our knowledge, **1** represents the most investigated iTMC emitter in an LEEC. The performances of all of the devices using **1** have been summarized in Table 2. The device architectures are somewhat more complex than the simplest reported LEECs, which themselves are single layer devices comprised of a neat film of emissive iTMCs sandwiched between a transparent conducting anode, usually indium tin oxide (ITO), and an air-stable cathode such as gold, silver, or aluminium. The ability to use air-stable, high-work-function electrodes is an advantageous characteristic of LEECs compared to OLEDs.

**Table 2:** Summary of LEECs reported employing **1**

Entry	Device config.	Bias	*t*_on_ (h)	*L*_max_ (cd m^−2^)	CE (cd A^−1^)	EQE (%)	PE (lm W^−1^)	*t*_1/2_ (h)	References
1	ITO/PEDOT:PSS/Ir/Al	3.0 V	70.2	219		2.2	6.1	668	[23]
2	ITO/PEDOT:PSS/Ir:IL (4:1)/Al	3.0 V	7.2	334		3.0	8.7	69	[23]
3	ITO/PEDOT:PSS/Ir:IL (1:1)/Al	3.0 V	0.7	375		5.6	16.3	7.8	[23]
4	ITO/PEDOT:PSS/Ir:[EMIM][PF_6_] (4:1)/Al	3.0 V	0.4^a^	497			4.6	81	[36]
5	ITO/PEDOT:PSS/Ir:[EMIM][PF_6_] (1:1)/Al	3.0 V	0.05^a^	615			4.9	4.1	[36]
6	ITO/PEDOT:PSS/Ir:IL (4:1)/Al	3.0 V	1.37^a^	269			8.6	103	[36]
7	ITO/PEDOT:PSS/Ir:IL (1:1)/Al	3.0 V	0.06^a^	586			16.7	4.3	[36]
8	ITO/PEDOT:PSS/Ir:[HMIM][PF_6_] (4:1)/Al	3.0 V	2.2^a^	153			5.6	134	[36]
9	ITO/PEDOT:PSS/Ir:[HMIM][PF_6_] (1:1)/Al	3.0 V	0.37^a^	302			14.1	5.5	[36]
10	ITO/PEDOT:PSS/Ir/Al	3.0 V	11.5^a^	219			6.1	668	[36]
11	ITO/PEDOT:PSS/Ir + 0.1wt% KPF_6_/LiF/Al	1.5 mA	63	1560	3.1	1.01	2.3	295	[37]
12	ITO/PEDOT:PSS/Ir + 0.1wt% LiPF_6_/LiF/Al	1.5 mA	4.6	4950	9.9	3.21	5.8	37	[37]
13	ITO/PEDOT:PSS/Ir + 0.33wt% LiPF_6_/LiF/Al	1.5 mA	0.003	3030	6.0	1.97	3.8	137	[37]
14	ITO/PEDOT:PSS/Ir + 0.1wt% NH_4_PF_6_/LiF/Al	1.5 mA	77	1410	2.8	0.92	2.2	199	[37]
15	ITO/PEDOT:PSS/Ir/LiF/Al	1.5 mA	49	80	2.4	0.77	1.9	167	[37]
16	ITO/PEDOT:PSS/Ir:IL (4:1)/Al	3.0 V	7.2	334	2.8	3.0	8.7	70	[21]
17	ITO/PEDOT:PSS/Ir:IL (1:1)/Al	3.0 V	0.7	375	7.1	5.6	16.3	7.8	[21]
18	ITO/PEDOT:PSS/Ir:IL (4:1)/Al	100 Am^−2^		ca. 900					[12]
19	ITO/PEDOT:PSS/Ir:IL (4:1) + trace Cl^−^/Al	100 Am^−2^		ca. 450					[12]
20	ITO/PEDOT:PSS/Ir:IL (3:1)/Al			ca. 260	10.0				[38]
21	ITO/PEDOT:PSS/Ir:IL (4:1)/Al	3.5 V at 293 K		Ca. 255	Ca 9.5				[39]
22	ITO/PEDOT:PSS/Ir:IL (4:1)/Al	3.5 V at 333 K		Ca. 850	Ca. 3.0				[39]

*L*_*max*_ is maximum luminance observed from device, *λ*_*EL*_ is electroluminescent emission maximum, *t*_*on*_ defined as time to reach maximum luminance, *CE* is current efficiency, *EQE* is external quantum efficiency, *PE* is power conversion efficiency, *t*_1/2_ is the time taken for the device luminance to fall to half the maximum value

^a^ *t*_on_ times defined as time to 100 cd m^−2^ luminance

Upon application of an external bias to the LEEC, there is a large initial barrier to charge injection. As migration of the ions in the emissive layers progresses, an electric double layer forms and the barrier to injection drops significantly until eventually charge injection at very low driving voltages (typically ca. 3 V) becomes facile. A charge-hopping mechanism ensues akin to that found in an OLED and emission is realized upon radiative decay of the formed exciton.

**Fig. 4:**
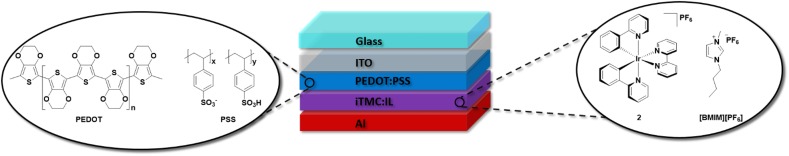
Typical device architecture of a LEEC employing **1** as the emissive layer

Although these single-layer devices readily generate light, various groups have shown in the last decade that small modifications to the device architecture can yield vastly improved LEEC performance. Figure 4 depicts these modifications in what can now be considered the most popular device architecture for LEECs, bearing two crucial features that differentiate it from the early reports. As mentioned, LEECs do not require charge-injecting layers to function, but nevertheless the ITO anode is invariably coated with PEDOT:PSS (an electrically conducting mixture of poly(3,4-ethylenedioxythiophene) and poly(styrenesulfonate)) since it facilitates the formation of uniform iTMC thin films on the ITO substrate and it improves hole injection. Devices fabricated in the absence of PEDOT:PSS are prone to forming crystalline-like domains within the film, which can have deleterious effects on the device performance and batch-to-batch reproducibility [33–35].

These two-layer devices nevertheless can give good performance, provided that the optoelectronic properties of the iTMC are also favorable. Entry 1 effectively demonstrates this principle with the device based on **1** exhibiting a remarkably long lifetime of 668 h. The authors attribute this stability to the relatively large calculated ^3^MC-T_1_ energy gap for **1** [23]. Theory and experimental observations have implicated ^3^MC states in an elongation of the N_pyridyl_-Ir bond of the C^N ligands of [Ir(C^N)_2_(N^N)]^+^ complexes, which accounts for the efficient non-radiative quenching resulting from these states [40]. In addition, work on ruthenium(II) complexes has suggested that this bond lengthening/breaking process within the ^3^MC state introduces a free coordination site that allows small molecules such as water to coordinate to the metal, quenching the emission and leading to degradation products within the device [13, 41]. Thus, devices employing complexes with a small ^3^MC-T_1_ energy gap tend to not be stable unlike the case with **1** where the device stability is enhanced. In addition, the reversible electrochemistry in **1** results in its capacity to act as an effective charge transport material and thereby resist electrochemical degradation processes that also impact device lifetimes.

Aside from the addition of PEDOT:PSS, an IL additive is also normally doped into the emissive layer to enhance charge mobility and to reduce turn-on times (*t*_on_, defined as the time taken to reach maximum luminance under constant bias) [42]. The most common IL used in LEECs is 1-butyl-3-methylimidazolium hexafluorophosphate, [BMIM][PF_6_]. Entries 1–3, Table 2, demonstrate the remarkable differences in LEEC performance produced by just varying the ratio of **1** and [BMIM][PF_6_] in an otherwise identical device configuration [23]. In the absence of IL, an extremely long *t*_on_ of more than 70 h is observed. This is attributed to low ionic mobility of the PF_6_^−^ anions, possibly due to the formation of microcrystalline domains in the film. Addition of IL circumvents this problem, improving ionic conductivity and thus charge transport in the device in addition to disrupting any possible crystallite formation. Entries 2 and 3, Table 2, show the performances of two devices with different weight ratios of iTMC to IL (4:1 and 1:1, respectively). Both devices show improved *t*_on_ times (7.2 and 0.7 h, respectively) as well as improved EQEs (3.0 and 5.6 %, respectively); where EQE is the external quantum efficiency, defined as the ratio of electrons injected into the device to photons outcoupled from the device. However, the addition of IL comes at the considerable cost of device stability as measured by its lifetime, *t*_1/2_ (*t*_1/2_, defined as the time taken for the device to reach half of its maximum luminance). In the absence of IL, this device lasts for up to 668 h, but lifetimes are dramatically reduced for the devices constituting iTMC:IL ratios of 4:1 (69 h) and 1:1 (7.8 h). This further reduction of device stability with increasing amounts of IL is representative of the behavior in LEECs, regardless of iTMC emitter, and illustrates the trade-off between *t*_1/2_ and *t*_on_ that has been a significant challenge to overcome. The compromise between attaining good device performance (EQEs, *t*_on_) and reasonable device lifetimes has meant the most popular iTMC:IL ratio employed has been 4:1 weight by weight.

Significant efforts have been expended to overcome this compromise in device performance. The most successful strategy is based on application of a pulsed current driving method in place of the more established constant voltage method and will be discussed multiple times in the following sections of this chapter. The majority of the remaining entries in Table 2 illustrate other approaches researchers have taken to tackle this issue, which will be discussed here.

Entries 4–10 summarize a study undertaken by Bolink et al. [36] to elucidate how using different ILs impact *t*_on_. It should be noted that in this study they define *t*_on_ as the time taken for the LEEC to reach 100 cd m^−2^, as opposed to maximum luminance in the device. The study uses ILs comprised of imidazolium cations of differing *N*-alkyl chain length: 1-ethyl-, 1-butyl- and 1-hexyl-3-methylimidazolium, each as their hexafluorophosphate salts. They demonstrated that the ethyl analogue, which is the most conducting IL, demonstrated the fastest *t*_on_ times (0.4 h for 4:1 iTMC:IL ratio). Although the *t*_1/2_ value was lowest for this IL, it was reasoned that the higher luminance values observed for this device were the major contributing factor for this shortened *t*_1/2_ and that actually the overall device stability had not been significantly impacted even when compared with the control device bearing no IL. Nevertheless, despite this study, [BMIM][PF_6_] is still the most popular choice of ionic liquid.

In a similar guise, entries 11–15 and Table 2, summarize the recent contribution by Slinker et al. [37] where they explored the effect on the response time of doping in inorganic salt additives. They reasoned that the large size of the iTMC cations renders the complexes to be essentially stationary in the device, meaning that upon initial application of a bias, the cation density at the cathode is initially much lower than the anion concentration at the anode, leading to an imbalance of charge injection into the device. However, by doping in small amounts of alkali metal cation hexafluorophosphate salts, they demonstrated that indeed fast *t*_on_ times under constant current conditions can be achieved, with the LiPF_6_ additive showing the best performance due to the small size of the Li^+^ cation. Crucially, this also led to more balanced charge injection that ultimately improved not just response times, but also luminance values and EQEs.

Aside from functioning as a useful standard for exploring new device physics, **1** also serves as a helpful reference compound for chemists to compare the performance of devices operating with new emitters. However, as alluded to above, reproducibility across devices can be a challenge. Entries 1 and 10, 2 and 16, and 3 and 17 reproduce each other well. However, comparison of entries 2 and 3 with the corresponding devices in entries 6 and 7 demonstrates considerable variation in device performance. The device in entry 6 in particular shows significant differences in the luminance (269 cd m^−2^) and *t*_1/2_ (103 h) values compared with its counterpart LEEC in entry 2 (334 cd m^−2^ and 69 h). Housecroft and co-workers have recently addressed this issue, demonstrating that the poor batch-to-batch reproducibility of these devices was attributable at least in part to trace Cl^−^ in the sample [12]. As outlined above, using silver salt-assisted syntheses they were able to isolate **1** with improved purity, leading to devices with superior luminance levels (ca. 900 cd m^−2^, entry 18) compared with those containing trace chloride impurities (ca 450 cd m^−2^, entry 19). High-purity samples are crucial for achieving good device performance, and indeed aside from the presence of Cl^−^, trace water has also been implicated in impacting device performances of ruthenium-based LEECs [13].

Entries 20-22 are beyond the scope of this discussion, with the relevant references discussing the device physics surrounding the peculiar operational mechanism of LEECs. They are included for the reader’s reference to illustrate additional examples reported where **1** has been the constituent emitter in the LEEC [38, 39].

## Blue

To date, attaining simultaneously efficient, stable, and deep-blue-emitting LEECs remains the most pressing issue for LEEC development. The challenge of obtaining high-performance blue-emitting devices is well-known for both organic and inorganic light-emitting devices, and while it has largely been addressed in the former case, this topic is still the source of very active research for OLEDs. For example, a recent report detailed the performance of a new champion blue OLED, which showed simultaneously deep-blue emission and high device efficiency (EQE = 10.1 %) [43]. However, this efficiency value still falls well below the efficiencies reported for red or green (EQE ~ 30 %), or even sky-blue OLEDs (EQE >20 %) [4].

In the case of LEECs, the situation is more dire. To date, no LEEC has even been reported emitting blue light close to the ‘ideal deep blue’ CIE coordinate (CIE: 0.15, 0.06, as defined by the European Broadcast Union, EBU), let alone with good efficiency. Furthermore, the stability of these sky-blue LEECs is demonstrably inferior than their OLED counterparts; device lifetimes are often in the tens of hours at best, compared with thousands of hours reported for yellow or orange LEECs. Given that blue is a necessity in attaining white light from a typical RGB color combination, overcoming this issue is of pressing concern. A summary of the relevant performance metrics of blue LEECs discussed herein is given in Table 3.

**Table 3:** Summary of LEECs employing blue-emitting iTMCs

Entry	Complex	*λ*_PL_ (nm)	*Φ*_PL_ (%)	*L*_max_ (cd m^−2^)	CE (cd A^−1^)	EQE (%)	PE (lm W^−1^)	*t*_1/2_ (h)	CIE (x)	CIE (y)	λ_EL_ (nm)	References
1	**3**	452, 480	20	39	0.65	0.28			0.20	0.28	460, 486	[44]
2	**3** ^*a*^	452, 480	20	23	0.51	0.21			0.33	0.45	460, 490, 526	[44]
3	**4** ^*a*^	492	100	8		14.4	32.1	2.17	0.20	0.36		[45]
4	**4** ^*b*^	493	40	1700		4.6	11		0.20	0.41	567	[33]
5	**5**	489	24	39	8.4	3.4			0.25	0.46	497	[46]
6	**6**	472, 490	54	15	18.3	7.6	18		0.22	0.41	474, 494	[47]
7	**7**	440	13	113	4.7		1.95	0.017	0.24	0.40	500	[48]
8	**8**	472, 501	0.001	15.4	0.2			24.3	0.43	0.53	560	[49, 50]
9	**9**	477, 500		16.1	0.6			29.8	0.41	0.53	556	[50]
10	**10**	480, 509	3	37	8.7			15.83	0.26	0.48	486, 512	[51]

*λ*_*PL*_ is the solution-state emission maximum in MeCN, *Φ*_*PL*_ is the photoluminescence quantum yield in deaerated solution, *L*_*max*_ is the maximum luminance observed from device, *CE* is the current efficiency, *PE* is the power conversion efficiency, *t*_*1/2*_ is the time taken for the device luminance to fall to half the maximum value, *λ*_*EL*_ is the electroluminescence emission maximum

^a^Measurement in DCM

^b^Measurement in 2-MeTHF

### Efficiency

**Fig. 5:**
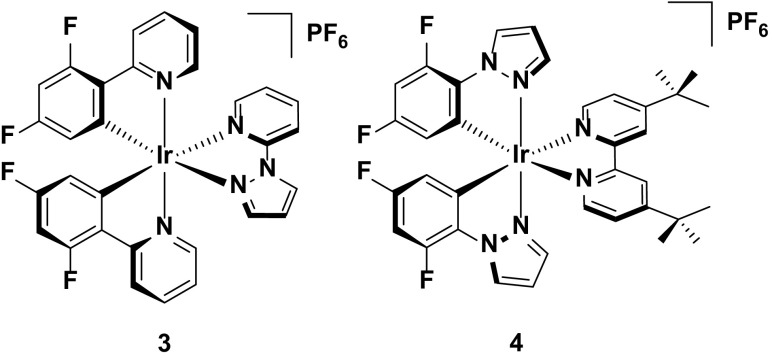
Blue-emitting iridium complexes bearing pyrazole-type ligands

The widely accepted paradigm to achieve blue emission requires that the HOMO be stabilized with electron-withdrawing groups located on the phenyl ring of the C^N ligands and the LUMO be destabilized with electron-donating groups located on the ancillary N^N ligand. The most commonly used electron-withdrawing groups used are fluorine atoms [52] with examples of complexes shown in Fig. 5. Further blue-shifting of the emission can be achieved by incorporating electron-rich heterocycles within the ligand frameworks of either the N^N (**3**) or C^N (**4**) ligands [33, 44, 45]. This strategy is most effective when this structural modification occurs within the ancillary ligand, as exemplified by the greater blue shift in emission observed for **3** (λ_PL_ = 451, 484 nm in MeCN) compared to **4** (λ_PL_ = 492 nm in DCM).

The blue emission in solution observed for **3** translates to its performance in the device, with CIE coordinates in the sky blue of (CIE: 0.20, 0.28). Despite being reported in 2008, this LEEC nevertheless remains the bluest reported for any iridium emitter to date. However, the emission observed for this device is still a long way from the ideal ‘deep-blue’ coordinates required in RGB devices (CIE 0.15, 0.06). Furthermore, this device functions in the absence of ionic liquid, which results in essentially impractical turn on times (*t*_on_ = 7.1 h). When an ionic liquid dopant is added, the response time shortens dramatically (*t*_on_ = 1.1 h) but the observed color is also greatly red-shifted (CIE 0.33, 0.45). Aside from the device based on **3** achieving the bluest emission reported to date, the performance of **3** in the device is relatively poor, with low efficiencies and brightness levels reported for both the IL free device (EQE = 0.28 %, *L*_max_ = 39 cd m^−2^) and IL-doped device (EQE = 0.21 %, *L*_max_ = 23 cd m^−2^).

By contrast, although complex **4** displays an emission profile that is strongly red-shifted compared to **3**, it is a much more efficient emitter in the device. Indeed, this high efficiency has made it a favored choice of emitter either for blue-emitting LEECs [30, 33, 53], or as the blue component in white LEECs [45, 54–56]. The performance of this emitter in white LEECs will be revisited in Sect. 7.

**Fig. 6:**
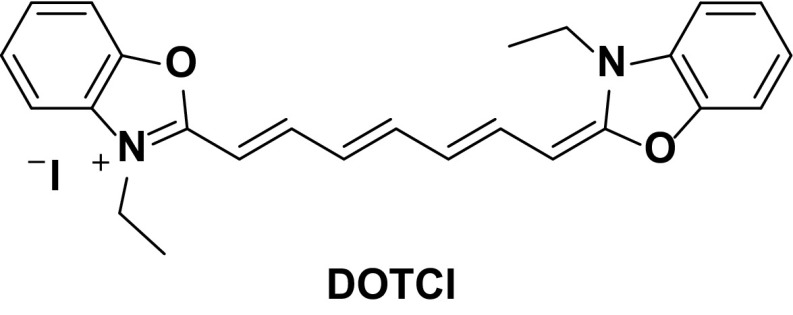
Organic dopant used by Wong to improve the efficiencies of **4**

As a blue/blue-green emitter, the device reported based on **4** displays extraordinarily high device efficiencies (EQE = 14.4 %, P. E. = 32.1 lm W^−1^). These values can vary significantly, depending on the device architecture. For example, the first reported LEEC employing this complex gave a comparably low overall EQE of 4.4 %, using a typical device architecture of ITO/PEDOT:PSS/Ir/Al. However, it is worth noting that this device displays very high brightness for a LEEC (entry 4, *L*_max_ = 1700 cd m^−2^) [33]. Since then, Wong in particular has explored different means by which charge injection and transport can be improved using this complex as an emitter. For example, it was shown that by doping small amounts (up to 1.0 wt %) of a pure organic NIR emitting laser dye, 3,3′-diethyl-2,2′-oxathiacarbocyanine iodide, **DOTCI**, (Fig. 6) into the emissive layer of complex **4**, higher device efficiencies could be obtained (EQE = 12.8 % for 0.01 wt % **DOTCI**) than without any dopant (EQE = 9.06 % for the pristine device **DOTCI**) [57]. The intrinsic hole transporting properties of **4** leads to the formation of the charge recombination zone near the cathode, which facilitates exciton quenching. This charge imbalance can be mitigated by doping in **DOTCI**. **DOTCI** has a much higher HOMO than **4** and therefore impedes hole transport but has a similar LUMO that thereby keeps electron mobility balanced. Furthermore, the poor spectral overlap between the emission of **4** and the absorption of **DOTCI** results in minimal quenching of the iridium-based emission by energy transfer to the guest, which would otherwise negatively impact the efficiency of the device.

**Fig. 7:**
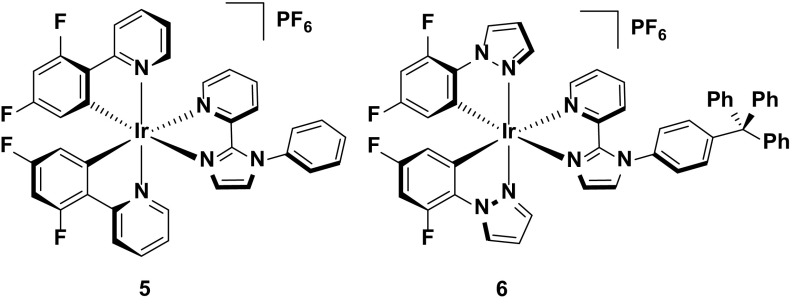
Iridium complexes bearing pyridylimidazole ancillary ligands

Aside from exploring dopants, Wong has also used **4** to study the effects of incorporation of additional layers to the LEEC architecture to further balance charge transport. The archetypal device based on **4** (ITO/PEDOT:PSS/**4**/Ag) gave reasonably good efficiencies (EQE = 8.5 %). However, the efficiency of the device could be further improved by incorporating a high work function cathode within the device (ITO/PEDOT:PSS/**4**/Ca/Ag, EQE = 9.6 %) and adding a hole injecting layer as well (ITO/PEDOT:PSS/TPD/**4**/Ca/Ag, EQE = 10.5 %). It should be noted that the strong hole transporting characteristics of **4** meant that addition of only the hole injecting layer actually impeded device efficiencies (ITO/PEDOT:PSS/TPD/**4**/Ag, EQE = 6.8 %), providing further evidence of the necessity of balancing charge injection and transport in the device (Fig. 7).

A different strategy that has been used to improve device efficiencies is by adding steric bulk to the complex to inhibit intermolecular quenching processes. Complexes **5** and **6** demonstrate this strategy. Both complexes are blue-green emitters in acetonitrile solution (*λ*_PL_ = 489 nm for **5** and 472, 490 nm for **6**), with **6** blue-shifted due to the additional pyrazole rings incorporated within the cyclometalating ligands. The most important structural difference between **5** and **6** is the presence of the trityl group on the ancillary ligand of **6**. This bulky unit serves to increase the molecular spacing between emissive molecules in the film, which leads to reduced excited-state self-quenching that negatively impacts the device efficiencies. Indeed, despite **6** being moderately blue-shifted both in solution and in the device (*λ*_EL_ = 474, 494 nm, CIE 0.22, 0.41) compared to **5** (*λ*_EL_ = 497 nm, CIE 0.25, 0.46), the LEEC with **6** shows greatly improved efficiencies (CE = 8.4 cd A^−1^ for **5** and 18.3 cd A^−1^ for **6**; EQE = 3.4 % for **5** and 7.6 % for **6**).

**Fig. 8:**
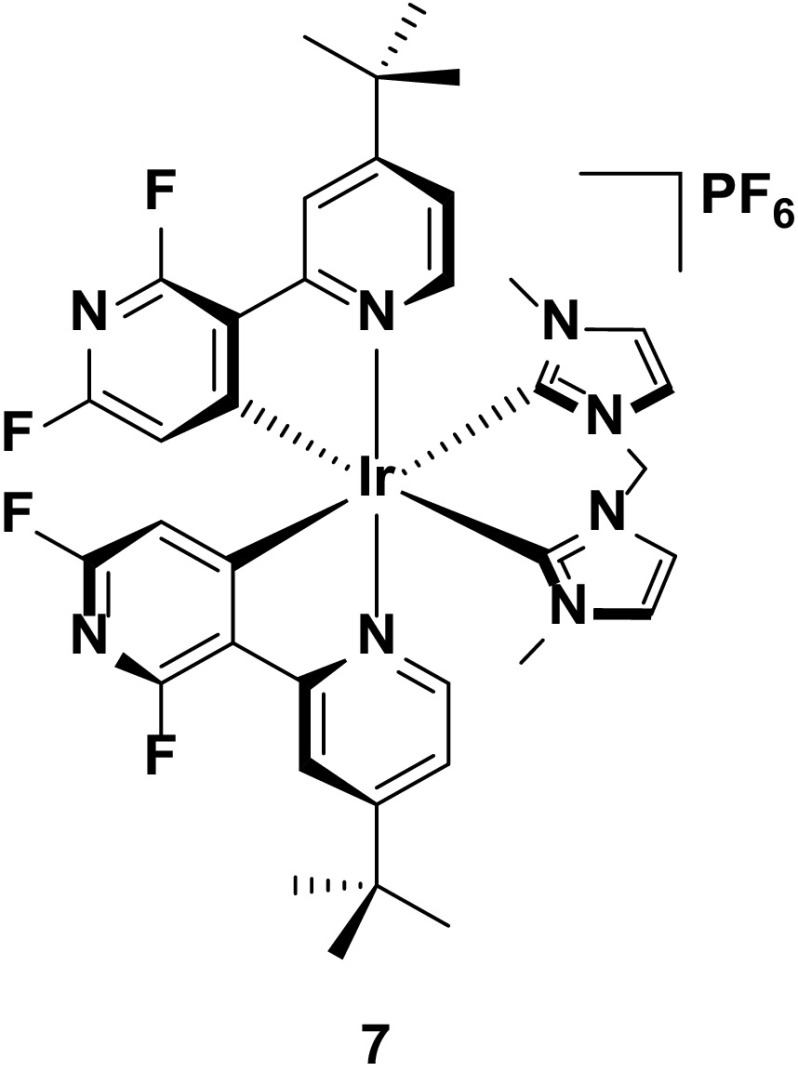
Blue-emitting iridium complex bearing an NHC ancillary ligand

Finally, complex **7** represents the unpredictability in designing new blue emitters for devices. For the ancillary ligand, this complex uses *N*-heterocylic carbenes (NHCs), which are very strongly σ-donating heterocycles that can strongly destabilize the LUMO, invoking a significant blue shift in the emission. The potency of these heterocycles is well known, with near-UV emission having been reported for iridium complexes containing multiple NHCs within the ligand frameworks [43, 58]. Furthermore, the cyclometalating ligand incorporates a nitrogen ring into the 5-position of the cyclometalating ring. This nitrogen acts as a strongly σ-withdrawing unit that serves to stabilize the HOMO in concert with the fluorine rings in the 4,6-positions. Thus, in solution, **7** is the bluest emitter reported to date to have been incorporated into a LEEC (*λ*_PL_ = 440 nm)—significantly bluer than complexes **3** to **6**. Unfortunately, for all the effort to blue shift the emission of this complex, ultimately the color of the device is red-shifted, even in comparison with the devices discussed above (*λ*_EL_ = 500 nm, CIE 0.24, 0.40) (Fig. 8).

### Stability

Although the examples above have demonstrated that high efficiencies are possible for sky-blue LEECs, the two most significant issues still to be addressed with these LEECs is their lack of deep-blue color and crucially their poor stability. There are many reasons for the poor stability of these devices, but one factor in particular thought to be contributing to poorer device performance is the presence of C_aryl_-F bonds on the cyclometalating ligands. It has been posited that the highly electron deficient C^N ligands make them susceptible to chemical degradation by nucleophilic aromatic substitution of the fluorine substituent. A study by Bolink and co-workers[16a] on the stability of fluorine-containing green-emitting iridium complexes will be outlined in more detail in Sect. 4, but it is worth noting here that they demonstrated that of the four complexes studied, the complex bearing four fluorine substituents was far less stable than others bearing just two fluorine atoms, providing indirect evidence that indeed (multiply) fluorinated aromatic rings can be implicated in the electrochemical degradation of the emitter in the device. Similar degradation processes are believed to be operative in OLEDs [59, 60], but the harsher environment in the emissive layer of a LEEC means that this effect is more pronounced in this class of electroluminescent device. Thus, there is interest in designing new emitters that emit blue light without the need for fluorine substituents that might negatively impact the stability. In addition, there is interest in adopting hydrophobic substituents within the ligand framework to impede nucleophiles from coordinating to the iridium center and quenching the emission. These two strategies are exemplified by complexes **8** and **9**, with **9** in particular representing an all-in-one effort to achieve blue emission without impacting the device stability.

Like **7**, complexes **8** and **9** use NHCs within the ancillary ligand to destabilize the LUMO of these complexes. Despite both complexes bearing just ppy as the C^N ligands, they are both blue-green emitters in acetonitrile solution (λ_PL_ = 472, 501 nm for **8** and 477, 500 nm for **9**), with emission strongly blue-shifted and more ligand-centered compared to **1** (*λ*_PL_ = 605 nm) [49, 50] thereby demonstrating the feasibility of blue-shifting emission without using fluorine.

**Fig. 9:**
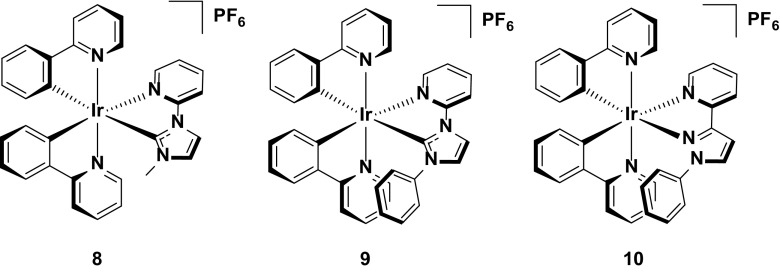
Iridium complexes bearing NHC ancillary ligands

Aside from the fluorine-free cyclometalating ligands, **9** also adopts the common intramolecular π-stacking strategy for improving the stability of the emitter in the device, utilizing a pendent phenyl substituent on the N^N ancillary ligand. This ring is predisposed to form an intramolecular π-stacking interaction with the phenyl ring of one of the C^N ligands, enveloping the iridium core in a supramolecularly caged hydrophobic scaffold that shields it from adventitious attack from prospective nucleophiles in the device that degrade the emitter [14, 61, 62]. This substitution pattern is more common for six-membered ring systems, such as the ligand 6-phenyl-2,2′-bipyridine (see Sect. 5 for examples), since the intramolecular π-stacking distance is usually shorter than in the case of five-membered rings such as the imidazolium ring in **9**, and is thus more effective at shielding the iridium core (Fig. 9).

The results of combining a fluorine-free ligand scaffold with an intramolecular π-stacking interaction do appear to improve the stability of the emitter. The devices with both **8** and **9** are much longer lived (*t*_1/2_ = 24.3 h for **8** and 29.8 h for **9**) than any of the LEECs with other blue/blue-green emitters discussed so far (e.g., *t*_1/2_ = 2.17 h for **4**), pointing to some extent to the merits of this strategy. However, it is important to note that the devices based on these materials are also greatly red-shifted. While they are blue-green in solution, the devices are essentially yellow-green (*λ*_EL_ = 560 nm for **8** and 556 nm for **9**; CIE: 0.43, 0.53 for **8** and 0.41, 0.53 for **9**), which is at least partly accountable for the improved device lifetimes. In addition, although there is a slight improvement in the device lifetime of the LEEC with **9** over that with **8**, this effect is not as pronounced as for some examples that will be discussed below. This is because the intramolecular π-stacking interaction in this complex is not as strong as it is for others based on 6-membered ring systems.

Finally, the use of complex **10**, a structurally related analogue of **9**, also attempts to combine strategies for improving stability with strategies for achieving blue emission. The ancillary ligand in this instance contains a pyrazole with coordination through the nitrogen. Complex **10**, like **9**, is fluorine-free and has an intramolecular π-stacking ring. This complex is red-shifted in MeCN solution compared to **8** or **9** (*λ*_PL_ = 480, 509 nm) but, surprisingly, is much bluer in the device, essentially retaining its solution-state emission characteristics (*λ*_EL_ = 486, 512 nm; CIE: 0.26, 0.48). The blue-shifted emission appears to impact the stability, however, with a lower device lifetime (*t*_1/2_ = 15.83 h) compared to the LEECs with **8** or **9**. The lower device stability for the device with **10** is probably due in part to its higher brightness compared to the LEECs with **8** or **9** (*L*_max_ = 15.4 cd m^−2^ for **8**, 16.1 cd m^−2^ for **9** and 37.0 cd m^−2^ for **10**). Nevertheless, although these complexes are the most stable among blue-green LEECs, none of them come close to commercially relevant stability requirements or even to some of the stability metrics reported for yellow or orange devices (thousands of hours).

## Green

Green emitters, like sky-blue emitters, have also been shown to achieve high efficiencies but relatively low stabilities. Many green-emitting complexes also contain fluorinated cyclometalating ligands, which certainly account for their shorter device lifetimes compared to yellow or orange LEECs. A summary of the emitters discussed in this section is given in Table 4.

**Table 4:** Summary of LEECs employing green-emitting iTMCs

Entry	Complex	*λ*_PL_ (nm)	*Φ*_PL_ (%)	*L*_max_ (cd m^−2^)	CE (cd A^−1^)	EQE (%)	PE (lm W^−1^)	*t*_1/2_ (h)	CIE (*x*)	CIE (*y*)	*λ*_EL_ (nm)	References
1	**11**	535	28	52		7.1	26.2	12	0.35	0.57	535	[63]
2	**12**	512	70	ca. 20	38	14.9	39.8	9	0.30	0.45	525	[64]
3	**13**			757	28.2	8.2	17.1	98	0.38	0.57	554	[65]
4	**13**	552	69	1028	9.8	2.85	5.2	59.8	0.38	0.57	554	[25, 65]
5	**14**	555	52	1066	9.8	2.92	5.4	48.3	0.39	0.56	558	[25]
6	**15**	555	59	1046	10.4	2.99	5.3	55	0.42	0.55	552	[25]
7	**16**	554	62	1095	9.6	2.90	5.3	13.2	0.39	0.55	555	[25]
8	**17**	515	41	837	6.4	1.95	3.08	0.01	0.31	0.57	ca. 540	[66]
9	**18**	548	32	157	5.7	2.3	8.6	223	0.44	0.55	555	[21]
10	**19**	559	54	190	6.1	2.2	7	356	0.47	0.52	570	[21]

*λ*_*PL*_ is the solution-state emission maximum in MeCN, *Φ*_*PL*_ is the photoluminescence quantum yield in deaerated solution, *L*_*max*_ is the maximum luminance observed from device, *CE* is the current efficiency, *PE* is the power conversion efficiency, *t*_*1/2*_ is the time taken for the device luminance to fall to half the maximum value, *λ*_*EL*_ is the electroluminescence emission maximum

### Efficiency

Most LEECs reported, even now, employ constant voltage-driving methods as a means of powering the device. Such a method results in generally slow turn-on times, but good performance metrics in terms of brightness and efficiency have been reported. Two of the best performing LEECs were reported employing complexes **11** and **12**. The crucial design feature of **11** is that it contains a bulky 4,5-diaza-9,9′-spirobifluorene ancillary ligand [63]. The bulk of this ligand ensures that intermolecular quenching in the solid state is minimized. The photoluminescence quantum yield in the neat film (*Φ*_PL_ = 31 %) is in fact not measurably different compared to the quantum yield in solution (*Φ*_PL_ = 28 %). Ultimately, it is this high neat film quantum yield that accounts for the very good device efficiency (EQE = 7.1 %; PE = 26.2 lm W^−1^). Similarly, complex **12** employs the bulky 4,4′-di-*tert*-butyl-2,2′-bipyridine ancillary ligand. In this case, the quantum yields are even higher (*Φ*_PL_ = 70 % in solution and 72 % in the film used for the device), giving device efficiencies that are extraordinarily high (EQE = 14.9 %) [64].

**Fig. 10:**
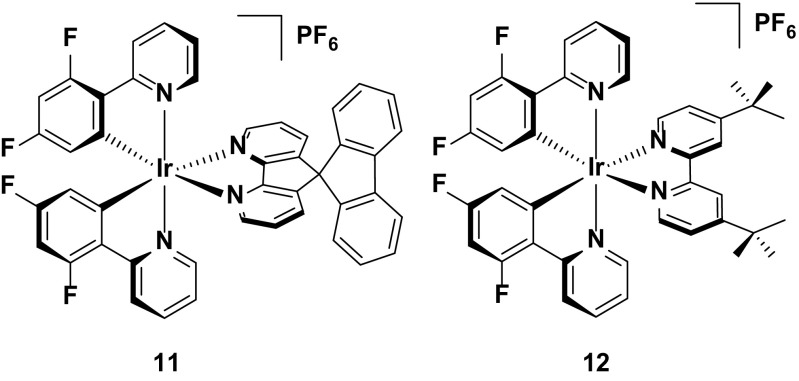
Efficient green-emitting devices based on constant voltage-driving conditions

Both of these devices are driven at a constant voltage (2.8 V for **11** and 3.0 V for **12**). This driving method leads to some drawbacks, including long device turn-on times (*t*_on_ = 1.5 h for **11** and 0.8 h for **12**) and also relatively poor stability (*t*_1/2_ = 12 h for **11** and 9 h for **12**) for both devices. However, it is worth noting that a pulsed current LEEC (which will be elaborated on below) based on **12** has been reported, demonstrating much lower efficiencies (EQE = 2.83 %) than reported under constant voltage [17] (Fig. 10).

In recent years an alternative driving method has become prevalent for operating these devices. Using a pulsed current driving method, high device efficiencies are also possible, but not at the expense of the stability or turn-on times of the devices. Arguably the champion green LEEC published to date is based on complex **13**, which uses a pulsed-current driving method to operate the device [65]. In the report, the authors explored driving the devices under a variety of different conditions, including varying the duty cycles from 25 % to 100 % (the latter of which correlates to operation under constant current) and also the average pulsed current density, from 18.75 to 150 A m^−2^. After much optimization, it was found that a 75 % duty cycle with a pulsed-current density of 25 A m^−2^ led to the best overall device performance. Crucially, it was found that high device efficiencies (EQE = 8.2 %, CE = 28.2 cd A^−1^, PE = 17.1 lm W^−1^) were possible, without adversely affecting the stability (*t*_1/2_ = 98 h) or the turn on time (*t*_on_ = 0.2 s). Indeed, these metrics make this LEEC the best overall performer, certainly when comparing the turn-on times and stabilities of the constant voltage LEECs employing **11** and **12**.

### Stability

**Fig. 11:**
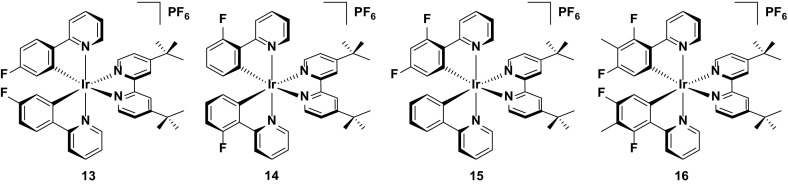
Multiply fluorinated green-emitting iridium complexes. The tetra-fluorinated complex displays much faster device degradation than the bis-fluorinated complexes

Complex **13** has also been reported as part of a larger study into the stability of iridium complexes bearing fluorinated C^N ligands.[16a] As identified in Sect. 3, such complexes are expected to be unstable, due to the reactivity of such aromatic rings bearing fluorine substituents. To unequivocally study this, Baranoff et al. synthesized complexes **13**–**16**, and studied their performance in the LEEC. All four complexes were designed to have similar photophysical properties (*λ*_PL_ = 552–555 nm, *Φ*_PL_ = 52–69 %) and similar device properties (EQE = 2.85–2.99 %, *L*_max_ = 1028–1095 cd m^−2^), such that the stability data would be directly comparable. They demonstrated that complex **16**, bearing four fluorine atoms, shows greatly reduced device lifetimes (*t*_1/2_ = 13.2 h) compared with the other three complexes (*t*_1/2_ = 48.3–59.8 h for complexes **13**–**15**). The device lifetimes for complexes **13**–**15** are by comparison rather long for green emitters; indeed, only **18** and **19** are longer lived green emitters in the device (Fig. 11).

To address issues with the stability of fluorine-containing iridium complexes, there has been interest in developing so-called fluorine-free ligands that are capable of achieving blue-shifted emission. Complex **17**, for example, was one of a number of complexes reported utilizing a 2,3′-bipyridine as the cyclometalating ligand to achieve a similar effect as that of the dFppy ligand, with the non-coordinating nitrogen acting to inductively withdraw electron density away from the metal centre, thereby stabilizing the HOMO energy and blue-shifting the emission as a result [66]. Although the stability of the devices based these emitters was not significantly improved (*t*_1/2_ = 0.01–2.5 h) it is worth noting that the CIE coordinates of **17** (CIE: 0.31, 0.57) are the closest to pure green (CIE 0.30, 0.60) that have been reported so far.

**Fig. 12:**
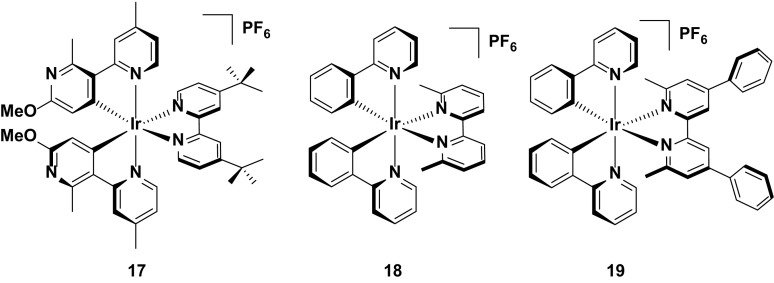
High stability green emitters

Complexes **18** and **19** provide a good comparison with **13**. The electroluminescence of these complexes is only slightly red-shifted (CIE 0.44, 0.55 for **18** and 0.47, 0.52 for **19**; *λ*_EL_ = 555 nm for **18** and 570 nm for **19**) compared to **13** (CIE 0.38, 0.57, *λ*_EL_ = 554 nm), but they show greatly improved stabilities (*t*_1/2_ = 98 h for **13**, 223 h for **18** and 356 h for **19**). This is attributed to the methyl groups in the *ortho*- position with respect to the pyridyl nitrogens, which act in a similar fashion to the intramolecularly π-stacking phenyl rings for **9** and **10**. It is plausible also that the lack of fluorine substituents appended to complexes **18** and **19** also adds to their stability in the device (Fig. 12).

## Yellow/orange

Moving from green emitters to yellow/orange leads to a pattern becoming apparent: as the color of the device shifts from blue to yellow the efficiency of the devices generally decreases, but the stability improves. Indeed, emitters of these colors surpass all others in terms of stability, with the most stable devices reported to date emitting in this color regime (Table 5).

**Table 5:** Summary of LEECs employing yellow/orange-emitting iTMCs

Entry	Complex	*λ*_PL_ (nm)	*Φ*_PL_ (%)	*L*_max_ (cd m^−2^)	CE (cd A^−1^)	EQE (%)	PE (lm W^−1^)	*t*_1/2_ (h)	CIE (*x*)	CIE (*y*)	*λ*_EL_ (nm)	References
1	**20**	588	9	395	13.2	6.1			0.53	0.47	588	[46]
2	**21**	595	7	284	14.7	6.1	15.3	660				[62]
3	**22**	605	23	16.45		9.16	26.91		0.51	0.48	580	[53, 63]
4	**23**	568	59	105		5.5	18.7				566	[47]
5	**24**	570	47	79		4.0	17.1				577	[67]
6	**25**	595	3	290	9.7	4.0	10.1	3000				[61]
7	**26**	595	3	70	3.1	1.1	3.3	1300				[68]
8	**27**	574	2	105	4.0			2000	0.49	0.50		[69]
9	**28**	623	26	130		0.3	0.46	110	0.55	0.44	594	[70]
10	**29**	593	5	183	8.2	3.4	8.6	950				[62]
11	**29**			650	3.6			>4000				[71]
12	**30**	583	43	684	6.5			2000	0.54	0.44	589	[72]
13	**31**	600	13	1024	3.5			2800				[14]
14	**32**	611	4	676	2.2			1204				[14]
15	**33**	645	2	261	0.7			>2800				[14]

*λ*_*PL*_ is the solution-state emission maximum in MeCN, *Φ*_*PL*_ is the photoluminescence quantum yield in deaerated solution, *L*_*max*_ is the maximum luminance observed from device, *CE* is the current efficiency, *PE* is the power conversion efficiency, *t*_1/2_ is the time taken for the device luminance to fall to half the maximum value, *λ*_*EL*_ is the electroluminescence emission maximum

### Efficiency

**Fig. 13:**
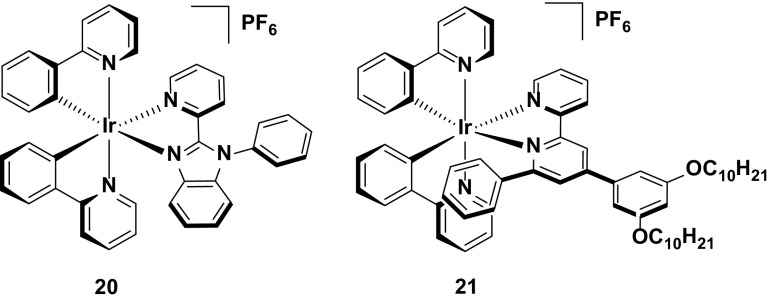
Yellow/orange emitters showing high device efficiencies

Efficiencies reported for yellow/orange LEECs tend not to be as high as for green LEECs, although several examples of complexes with comparable efficiencies have been reported. For example, the external quantum efficiencies reported for the devices using **20** and **21** are two of the highest (EQE = 6.1 % for both **20** and **21**) reported for this color to date [46, 62]. The origin of the high efficiency for **20** is not explained. In solution this complex is not especially emissive (*Φ*_PL_ = 9 % in MeCN) and no thin film PL data is reported. Complex **21** is also poorly emissive in solution (*Φ*_PL_ = 7 % in MeCN) but in this instance the value reported for the Φ_PL_ in the film is much higher (*Φ*_PL_ = 47 % in a film of iridium complex and [BMIM][PF_6_] in 4:1 molar ratio), which accounts for the good device performance. As with other complexes previously discussed, the performance of **21** in the device is attributed to the presence of the bulky hydrophobic substituents on the complex, which contribute to decreased quenching of the excitons formed in the device. An added benefit of the substitutions on the ancillary bipyridine ligand is that they improve the stability of the emitter in the device, with a very good device lifetime compared to many other LEECs reported in the literature (*t*_1/2_ = 660 h). This result is in contrast with the LEECs using complexes **9** and **10**, wherein the shielding of the iridium centre by the intramolecular π-stacking interaction was mitigated somewhat by the use of five-membered pyrazole and imidazolium rings (Fig. 13).

The highest efficiency yellow/orange device reported to date is complex **22**, which is the fluorine-free analogue of complex **11**, using the same 4,5-diaza-9,9′-spirobifluorene as the diimine ligand [53, 63]. Steric bulk of this ligand remains an important factor in preventing intermolecular quenching in the device by increasing the spacing between the chromophores, leading to a reasonably bright emitter in solution (*Φ*_PL_ = 23 %) and solid state (*Φ*_PL_ = 33 % in the ‘LEEC’ film containing the iridium complex and [BMIM][PF_6_] in a 1.3:1.0 molar ratio). Its initial device efficiency was reported to be 7.1 % using a simple LEEC architecture [ITO/Ir:[BMIM][PF_6_] (1.3:1.0 molar ratio)/Ag]. However, studies on improving the carrier injection efficiency of the device have since led to a record quantum efficiency (EQE = 9.16 %) reported for a yellow/orange device, based on a related device architecture [ITO/PEDOT:PSS/Ir:[BMIM][PF_6_]/Ag. This improved performance is likely to be due to the fact that **22** has preferred electron-transporting characteristics [53], and thus PEDOT:PSS, which is effective as a hole injecting layer, helps to balance charge transport in the device.

**Fig. 14:**
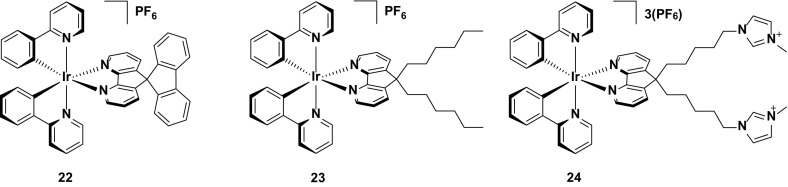
High efficiency yellow/orange emitter (**22**) and related complexes (**23** and **24**)

It is curious to note however, that analogues of **22**, using the same diazafluorenyl-type ligand (**23** and **24**) are in fact brighter in solution than **22** (*Φ*_PL_ = 59 % and 47 %, respectively), but display poorer performance in the device [67]. These emitters were designed to explore strategies for improving the turn-on time of the LEEC, with the charged groups appended to **24** anticipated to increase the rate of ion separation in the emissive layer similar to that previously demonstrated by Zysman-Colman et al. [73]. and thus more quickly lower the barrier to charge injection into the device. This effect is achieved (*t*_on_ = 1.1 h for **23** and 0.2 h for **24**) but at the detriment of the performance of the emitter in the device (EQE = 5.5 % for **23** and 4.0 % for **24**), suggesting that even minor changes to functionality peripheral to the electronics of the emitter can nevertheless have a significant effect on the efficiency (Fig. 14).

### Stability

**Fig. 15:**
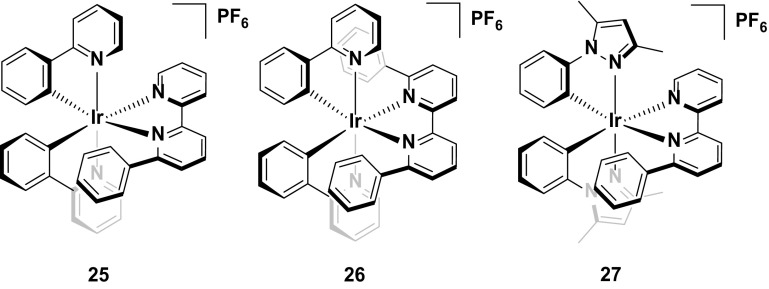
Champion stable LEECs operated at constant voltage

Complex **25** is the first example reported of a complex containing the intramolecular π-stacking motif alluded to previously. Within **25**, there is a short centroid-to-centroid distance of 3.48 Å between the phenyl ring on the bpy and one of the cyclometalating phenyl rings. This tight interaction maintains the structural integrity of the inner coordination sphere, even when the anti-bonding e.g., orbitals of the MC states are populated, and thus inhibits potential nucleophiles from coordinating to the metal centre upon population of the ^3^MC states. The devices reporting this emitter were operated under constant voltage and two values have been reported for the device lifetime based on this emitter (*t*_1/2_ = ca. 1300 h [36, 62, 68] or 3000 h [61]), with the longer value resulting from operating the device with a pre-biasing method (Fig. 15).

Although the intramolecular π-stack is an effective strategy for increasing device stability, it does have limits: complex **26**, with two incorporated π-stacking phenyl rings on the ancillary ligand results in poorer device performance compared to that with **25** [68]. Devices based on **25** and **26** both show long lifetimes (*t*_1/2_ = 1300 h for **25** and **26**). However, the luminance values of **25** (*L*_max_ = 110 cd m^−2^) are higher than for **26** (*L*_max_ = 70 cd m^−2^). Devices with different luminance levels are not necessarily comparable in terms of stability, since brighter devices intrinsically degrade more quickly. Thus, to compare devices of different luminance levels it has been argued that considering the total photon flux emitted from the device once the luminance reaches 1/5 of the maximum value, $E_{{t_{1/5} }}$ , is a more accurate assessment of its stability. In this instance, **25** showed higher $E_{{t_{1/5} }}$ values (13.6 J) than **26** (6.9 J), and thus it was concluded to be the more stable emitter. It was rationalized that the although the additional π-stacking ring further shields the metal centre, in order to maximize the dual π-stacking interaction there is a distortion of the inner coordination the sphere of the complex. This distortion in turn makes the MC states more thermally accessible, thus promoting exciton quenching and making the complex more susceptible to degradation reactions in the device.

**Fig. 16:**
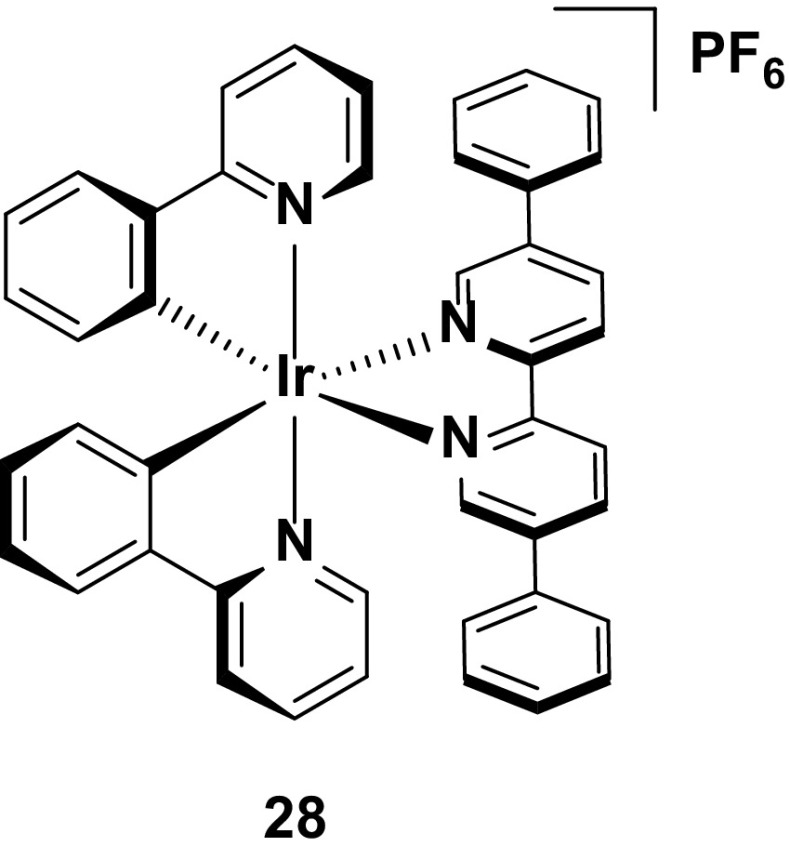
Stable LEEC based on a hydrophobic iridium complex

An alternative strategy designed to protect the iridium from adventitious attack of small molecule nucleophiles is shown for complex **27** [69]. Here, the methyl groups appended to the pyrazole rings add an additional steric shield to the metal centre similar to complexes **18** and **19**. This strategy confers excellent stability to the LEEC with the device with **27** showing higher lifetimes (*t*_1/2_ = 2000 h) than that reported using **25** as the reference emitter (*t*_1/2_ = 1290 h) (Fig. 16).

In general, hydrophobic substituents appear to improve the stability of the emitter in the device. For example, the phenyl rings on the 5,5′-positions of the ancillary ligand of complex **28** do not form an intramolecular π-stacking motif. Nevertheless, the hydrophobicity of these rings appear to confer good stability to the device, with good brightness (*L*_max_ = 130 cd m^−2^) and a reasonable device lifetime (*t*_1/2_ = 110 h) [70] without impacting greatly the emission color compared to **1**.

Given the aforementioned benefits of pulsed current LEECs, it is plausible that pulsed current LEECs based on complexes **25**–**28** would perform even better. Complex **29**, which is the methoxy analogue of **21**, is a good example of the contrasting performances of an emitter in a LEEC under constant voltage and pulsed current conditions. Under constant driving voltage, the device was reported to show good stability (*t*_1/2_ = 950 h) [62], but this is well below the value reported for this emitter under pulsed current conditions (*t*_1/2_ = 4000 h) [71]. Indeed, this latter lifetime is the longest reported for any iridium-based LEEC to date. The long lifetime in the device is coupled with higher brightness (*L*_max_ = 650 cd m^−2^ under pulsed current *vs.* 183 cd m^−2^ under constant voltage), although the efficiency under constant voltage is higher (CE = 8.2 cd A^−1^ under constant voltage versus 3.6 cd A^−1^ for pulsed current).

**Fig. 17:**
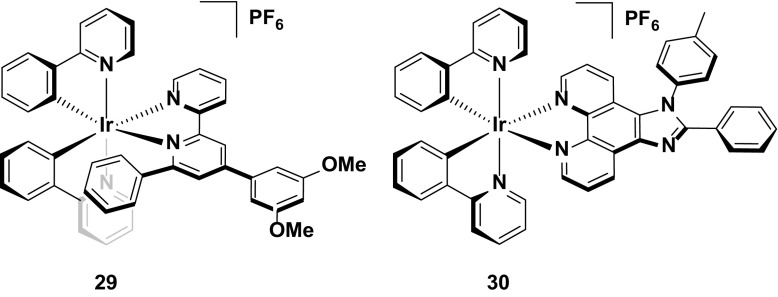
High stability emitters operated under pulsed-current conditions

The merits of the pulsed current driving method are exemplified by the device with **30**. To the best of our knowledge, this complex is the only emitter reported with a device lifetime of greater than one thousand hours (*t*_1/2_ = 2000 h) that does not have an intramolecular π-stacking motif. The device with this complex also shows a higher efficiency than that with **29** (CE = 6.5 cd A^−1^) under pulsed current driving [72] (Fig. 17).

Finally, complex **31** represents arguably the most stable emitter reported to date [14]. The values reported for complexes **31**–**33** are from operating the devices at an exceptionally high current density of 300 A m^−2^. Typically, pulsed-current LEECs are operated at average current densities of 50–100 A m^−2^ (**29** was operated at an average of 185 A m^−2^ and **30** at 100 A m^−2^); however, at these current densities no discernible degradation of the devices could be observed and thus a much higher average current density was required. The high stability of the device at these current densities for all three complexes (*t*_1/2_ = 2800 h for **31**, 1204 h for **32**, and >2800 h for **33**) is attributed to the silver-assisted synthesis of these complexes to ensure they are free of chloride impurities, which as discussed above, are detrimental to the performance of these complexes in the device.

**Fig. 18:**
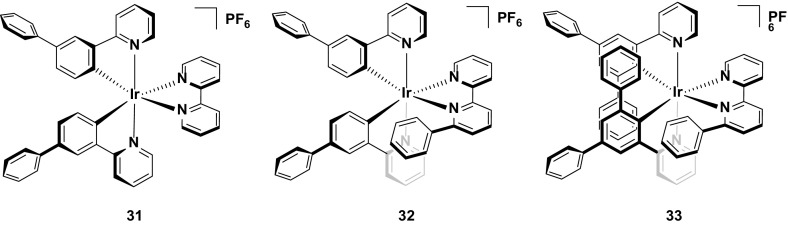
Champion-pulsed current LEEC for stability (**31**) and related analogues (**32** and **33**)

Since no E_t1/5_ values are reported it is difficult to discern which of the three complexes is the most stable; however, the much higher luminance value for **31** (*L*_max_ = 1024 cd m^−2^) compared with **32** (*L*_max_ = 676 cd m^−2^) and **33** (*L*_max_ = 261 cd m^−2^) suggest that it is the champion emitter in terms of device stability. It is surprising however, that **31** is the simplest of these structures, with no intramolecular π-stacking motif. These results illustrate that there are still challenges for correlating the structure of an emitter to its performance in the device (Fig. 18).

## Red

Although the challenge of designing blue emitters is still the greatest for LEECs (no LEEC so far has been reported to even achieve deep-blue emission, let alone with good device performance), only a small number of LEECs have been reported to have CIE coordinates close to the ideal red value (CIE 0.66, 0.33) and, like the blue LEECs reported to date, these devices all show poorer stability compared with yellow/orange LEECs (Table 6).

**Table 6:** Summary of LEECs employing red-emitting iTMCs

Entry	Complex	*λ*_PL_ (nm)	*Φ*_PL_ (%)	*L*_max_ (cd m^−2^)	CE (cd A^−1^)	EQE (%)	PE (lm W^−1^)	*t*_1/2_ (h)	CIE (*x*)	CIE (*y*)	*λ*_EL_ (nm)	References
1	**34**	624	0.68	7500		7.4	10		0.67	0.32	635	[33]
2	**35**	627	3	70	1.6	2.6			0.66	0.33	650	[46]
3	**36**	556	24	154	13.1	9.51		8.17	0.59	0.40	624	[24]
4	**37**	573	58	217	6.29	2.74		9.83	0.50	0.41	616	[24]
5	**38**	619	55		2.5	3.27	2.56		0.65	0.34	644	[74]
6	**39**	666	2.6	14		0.08		6.3	0.68	0.33	666	[75]
7	**40**	608	6	626	2.7	1.67		25	0.59	0.41	607	[76]
8	**41**	687	2	ca. 35	0.83		0.87	0.52	0.71	0.28	630	[77]
9	**42**	687	1	ca. 35	0.68		0.71	37	0.69	0.29	660	[77]

*λ*_PL_ is the solution-state emission maximum in MeCN, *Φ*_PL_ is the photoluminescence quantum yield in deaerated solution, *L*_max_ is the maximum luminance observed from device, *CE* is the current efficiency, *PE* is the power conversion efficiency, *t*_1/2_ is the time taken for the device luminance to fall to half the maximum value, *λ*_EL_ is the electroluminescence emission maximum

### Efficiency

**Fig. 19:**
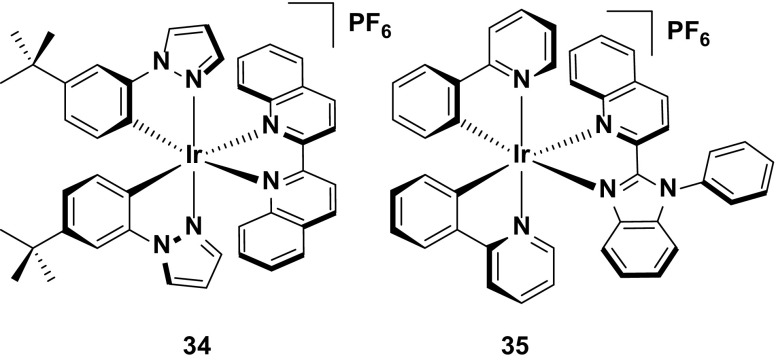
High efficiency, red-emitting iridium complexes

Although heterocycles such as pyrazoles and imidazoles are rarely used for red emission (due to their strong σ-donating character and their tendency to induce blue-shifting in the emission compared with pyridyl rings), two of the best red-emitting devices nevertheless utilize such heterocycles. Complex **34** utilizes a phenylpyrazole-type cyclometalating ligand but compensates for its blue-shifting effect by incorporation of the highly conjugated 2,2′-biquinoline ancillary ligand to red shift the emission [33]. Similarly, the LUMO destabilizing capabilities of the imidazole ring contained within the ancillary ligand of **35** are also compensated by the annelated benzene to form the benzimidazole and by the appended quinoline ring [46]. Both of these complexes are red emitters (*λ*_PL_ = 624 nm for **34** and 627 nm for **35**) in MeCN solution but they are only poorly emissive (*Φ*_PL_ = 0.68 % for **34** and 3 % for **35**), presumably as a function of the energy gap law. Photoluminescence quantum yield data in the solid state is not reported for either of these emitters so it is not possible to correlate these to the device performances. However, high efficiencies are reported for both devices, particularly the device based on **34**, (EQE = 7.4 % for **34** and 2.6 % for **35**), making them among the best red devices reported to date. In addition, the color of both devices essentially coincides with the pure red CIE coordinate (CIE 0.67, 0.32 for **34** and 0.66, 0.33 for **35** (Fig. 19).

**Fig. 20:**
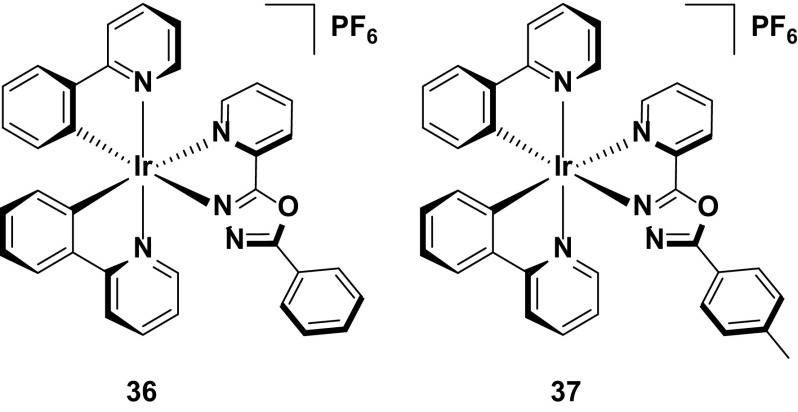
Champion red-emitting device (**36**) and its analogue (**37**)

One of the intrinsic issues with blue emitters is that the emission is frequently red-shifted in the device. For red emitters, this feature can act as an advantage, exemplified by complexes **36** and **37** [24]. In solution these complexes emit yellow light (*λ*_PL_ = 573 nm for **36** and 556 nm for **37**), but in neat film (*λ*_PL_ = 627 nm for **36** and 625 nm for **37**) and in the device (*λ*_EL_ = 624 nm for **36** and 616 nm for **37**) the emission is strongly red-shifted. The authors attribute this red shift to the possible formation of excimers in the condensed phase, due to strong π–π intermolecular stacking interactions observed in the crystal structures of **36** and **37**. Crucially, this red shift in emission observed in the LEEC is accompanied with impressive device performance, particularly for **36**, which shows the highest device efficiency of any red or yellow/orange device reported to date (EQE = 9.51 % for **36** and 2.74 % for **37**). This high efficiency certainly qualifies **36** as the champion red-emitting device reported to date. However, it is worth noting that the CIE coordinates of these devices (CIE 0.59, 0.40 for **36** and 0.50, 0.41 for **37**) are blue-shifted compared to the pure red CIE coordinates (CIE: 0.66, 0.33) required for RGB color coordinates (Fig. 20).

Finally, complex **38** is an interesting red emitter [74]. The extended conjugation of the perylenediimide unit was used to achieve deep-red emission, while also functioning as an electron-transporting moiety to balance the hole conducting capabilities of the iridium component. It was found that the iridium essentially does not contribute to the photophysics of the compound. The short emission lifetime (*τ*_e_ = 3.0 ns) and high quantum yield (*Φ*_PL_ = 55 %) for a red emitter (*λ*_PL_ = 619 nm) point instead towards fluorescence directly from the perylenediimide chromophore, an assignment supported by theoretical calculations that implicated only the perylenediimide unit in the electronics of the HOMO or LUMO. Thus, the iridium in this case acts only as an appended charged unit to enable this complex to function in the LEEC.

**Fig. 21:**
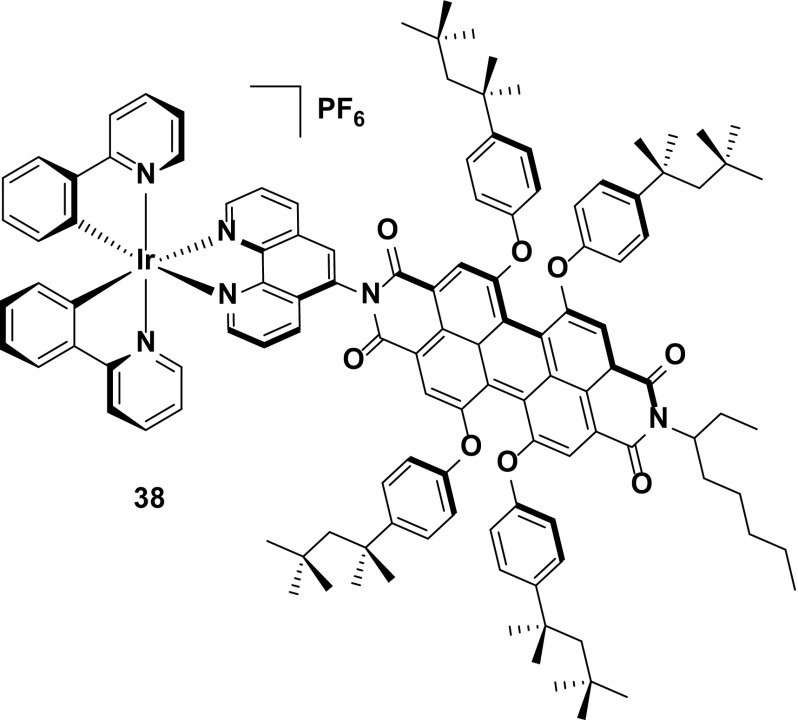
Deep-red-emitting iridium complex with good device efficiency

Crucially, the short emission lifetime is suggested to help in circumventing non-radiative quenching pathways in which typical triplet emitters are susceptible, leading to a good efficiency (EQE = 3.27 %) for a red-emitting device (CIE 0.65, 0.34). Although a useful feature, it is unclear if this compound is purely a singlet emitter, or whether it is in fact harvesting triplets as well, an important feature of typical phosphorescent iridium complexes. In this instance, it is possible that this strategy is in fact wasting the triplets generated in the emissive layer, defeating the object of utilizing an iridium-based material in the first place (Fig. 21).

### Stability

**Fig. 22:**
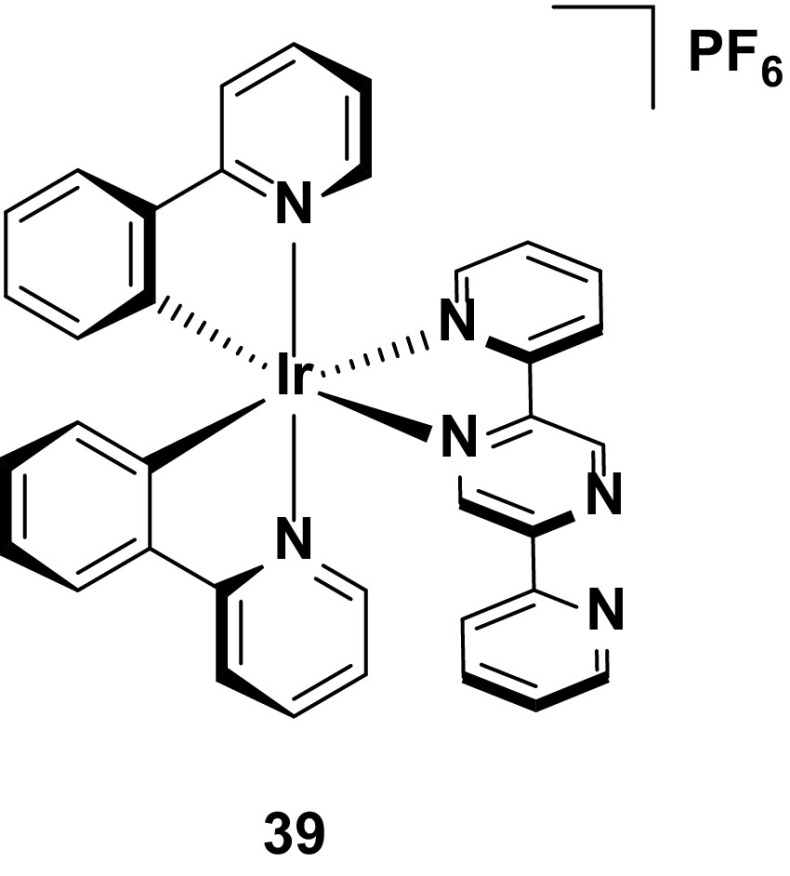
Iridium complex with good stability for a deep-red LEEC

Complex **39** employs a 2,5-dipyridyl(pyrazine) ancillary ligand which, demonstrating the opposite trend to complex **17**, shows red-shifted emission as a result of the electron-withdrawing nature of the non-coordinating nitrogen on the pyrazine ring [75]. Thus, in this instance, the LUMO is strongly stabilized. Further narrowing of the HOMO–LUMO gap comes by way of the non-coordinating pyridyl ring, which extends the conjugation on the ancillary ligand, red-shifting the emission further, both in solution (*λ*_PL_ = 666 nm) and the device (CIE 0.68, 0.33; *λ*_EL_ = 666 nm). Although the efficiency of the device is low (EQE = 0.08 %), the lifetime (*t*_1/2_ = 6.3 h) is rather long for a deep-red-emitting device. As with the examples below, much shorter device lifetimes compared with yellow/orange seems to be a general feature of deep-red-emitting LEECs (Fig. 22).

**Fig. 23:**
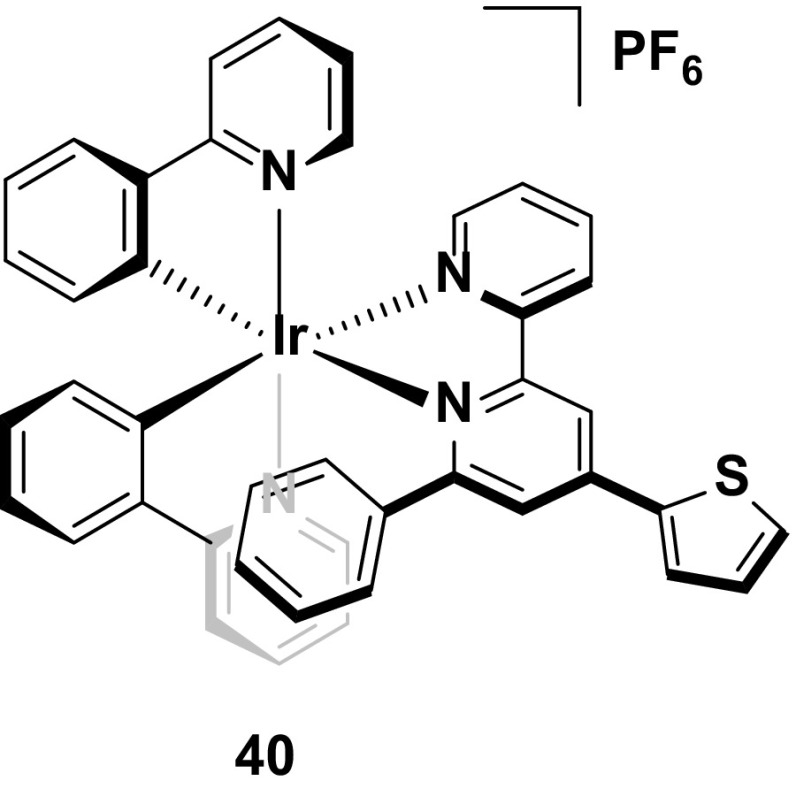
Intramolecularly π-stacked deep-red-emitting iridium complex

A number of red emitting intramolecularly π-stacked complexes have been reported but few of them demonstrate any appreciable stability when compared to their yellow/orange analogues. Indeed, even in the case of the most stable of the red-emitting complex bearing an intramolecularly π-stacking motif, **40**, the device lifetime (*t*_1/2_ = 25 h) is still very short compared with many other devices employing intramolecularly π-stacked complexes [76]. Although the luminance levels for this device are good (*L*_max_ = 626 cd m^−2^), the yellow-emitting devices utilizing structurally related complexes **32** and **33** are brighter and of course significantly longer lived. Clearly the stability of deep-red emitters is still lagging some way behind other devices reported to date (Fig. 23).

**Fig. 24:**
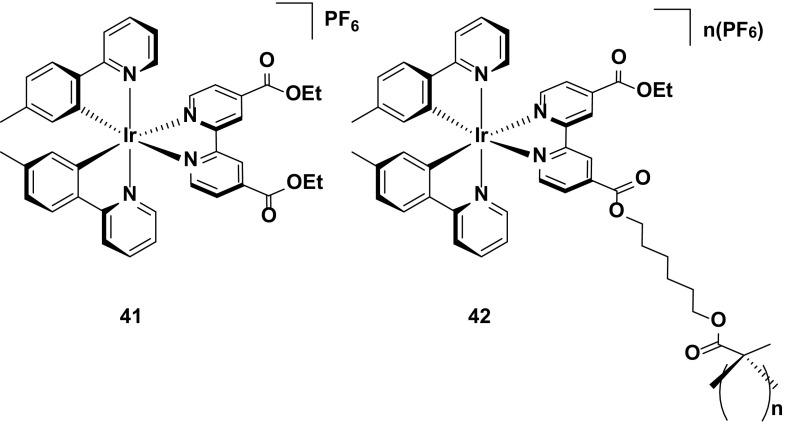
Deep-red-emitting iridium complex and its corresponding polymer, which shows greatly enhanced stability

An alternative strategy reported for improving the stability of this class of emitters is that shown in a comparative study between complexes **41** and **42**. By covalently tethering the emitter to a polymer backbone, it was suggested that this should lead to a more uniform distribution of the complex within the emissive layer, reducing aggregate formation and increasing the spatial distribution of the emitters within the device, thereby improving the stability. Indeed, this appears to be the case, with a significant enhancement in the device lifetime with **42** (*t*_1/2_ = 37 h) compared to the device with **41** (*t*_1/2_ = 0.52 h). Although in absolute terms the lifetime of **42** is still poor, it is nevertheless the longest of any red device (CIE 0.69, 0.29) reported to date, suggesting that this is a viable, underexplored strategy for improving the stability of the device, as well as highlighting similar challenges in achieving stable red LEECs as discussed for achieving stable blue LEECs (Fig. 24).

## White

Given the paucity of charged blue emitters, examples of white LEECs in the literature are subsequently scarce. Such devices broadly fall into one of two categories: host–guest systems, where a blue emitting host material is doped with a red emitter, or a multi-stack device where multiple emissive layers are combined within a single device to achieve white light. Both families of devices will be discussed.

Although in these publications it is common for multiple configurations to be reported, we will discuss only the devices in a particular report that demonstrate CIE coordinates closest to the ideal white coordinate (CIE 0.33, 0.33), which is the ultimate goal of white LEECs, regardless of the performance of other devices in the report. Likewise, often these devices are operated at multiple biases, leading to significant changes in the color and the performance. Again we will consider only the driving voltage/current that achieves the ‘whitest’ emission. The values are summarized in Table 7.

**Table 7:** Summary of white LEECs

Entry	Bias/V	*L*_max_ (cd m^−2^)	CE (cd A^−1^)	EQE (%)	PE (lm W^−1^)	*t*_1/2_ (h)	CIE (*x*)	CIE (*y*)	*λ*_EL_ (nm)	CRI	References
1	3.3	43	5.8	3.3	5.5	0.4	0.35	0.39	488, 612	80	[78]
2	3.5	31	11.2	5.2	10		0.40	0.44	497, 590	81	[46]
3	3.2	7.9	11.4	5.6			0.37	0.41	495, 600	80	[47]
4	3.6	7.2		7.3	13.5	2.83	0.33	0.33	Ca. 480, 550, 600		[55]
5	3.3	20.2	13.4	6.3	12.8	0.62	0.32	0.43	Ca. 500, 600	70	[56]
6	3.7	32		12.5	27	0.17	time dependent		Ca. 500, 600		[45, 54]
7	7.0	Ca. 400	0.41				0.32	0.34	475, 580		[79]
8	100 A m^−2^	845	8.5				0.38	0.47			[80]

*L*_*max*_ is the maximum luminance observed from device, *CE* is the current efficiency, *PE* is the power conversion efficiency, *t*_1/2_ is the time taken for the device luminance to fall to half the maximum value, *λ*_EL_ is the electroluminescence emission maximum, *CRI* is the color rendering index value of the device

### Host–guest LEECs

**Fig. 25:**
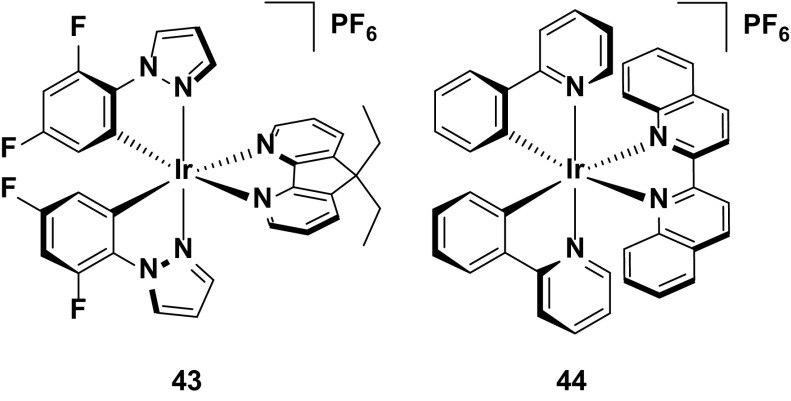
Host-guest LEEC containing **43** as the blue/green host component and **44** as the red dopant. Device: ITO/**43** (80.5 wt %), **44** (0.4 wt %), [BMIM][PF_6_] (19.1 wt %)/Ag, entry 1

The typical host–guest device is that based on **43**, a blue-green emitter (*λ*_PL_ = 491 nm in the neat film), and **44**, which is a deep-red emitter (*λ*_PL_ = 672 nm in the neat film) [78]. A small amount of **44** (0.4 wt %) doped into **43** ensures a partial energy transfer (although not stated in this case, other references on white emitting LEECs describe this kind of energy transfer as Förster in nature) [55] from the excited blue host to the red emitting guest, resulting in emission from both chromophores, and giving white light as a result. Despite being the first reported example of a white light-emitting LEEC based on a host–guest system, the device showed decent efficiencies (EQE = 3.3 %), and crucially the color of the device was very close to the ideal white point (CIE 0.35. 0.39) (Fig. 25).

**Fig. 26:**
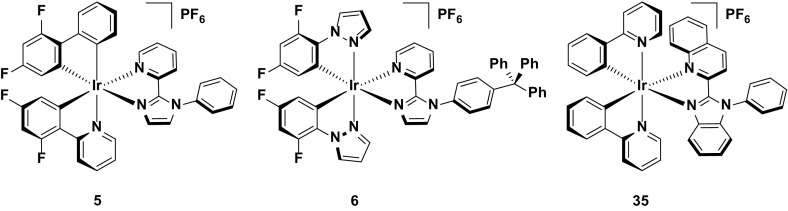
Host-guest LEECs containing **5** or **6** as the blue/green host component and **35** as the red dopant. Device: ITO/PEDOT:PSS/**5**: [BMIM][PF_6_]: **35** (1: 0.35: 0.002 molar ratio)/Al, entry 2. Device: ITO/PEDOT:PSS/**6**: [BMIM][PF_6_]: **35** (1: 1: 0.008 molar ratio)/Al, entry 3

An improvement in the device performance of the white LEEC is documented in entries 2 and 3 in Table 7. A device utilizing **5** as the host and **35** as the guest (1: 0.002 molar ratio) achieved greatly improved efficiencies compared to entry 1 (EQE = 5.2 %, CE = 11.2 cd A^−1^) [46], but with CIE coordinates that are modestly red-shifted (CIE: 0.40, 0.44) from the white point. Changing the host from **5** to **6** achieves a small increase in the efficiency (EQE = 5.6 %, CE = 11.4 cd A^−1^) and a slight blue shift in the CIE coordinates (CIE 0.37, 0.41) [47]. However, the brightness of the device based on **6** (*L*_max_ = 7.9 cd m^−2^) is greatly diminished compared with **5** (*L*_max_ = 31 cd m^−2^) (Fig. 26).

**Fig. 27:**
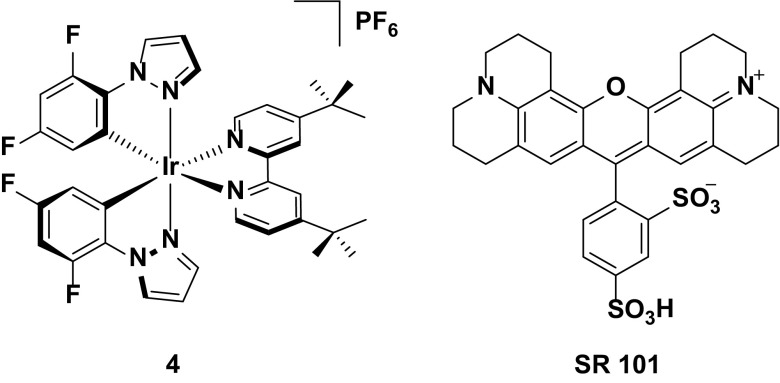
Host-guest LEEC containing **4** as the blue/green host component and **SR 101** as the red dopant. Device: ITO/PEDOT:PSS/**4** (79.5 wt %), **SR 101** (0.5 wt %), [BMIM][PF_6_] (20 wt %)/Ag, entry 4

The most commonly used donor complex in host–guest LEEC systems is **4** and these devices tend to offer improved performance (entry 4). The guest in the LEEC need not be an iridium complex. For instance, the device shown in entry 4 has an EQE of 7.3 % and employs a highly emissive (*Φ*_PL_ = 90 % in ethanol) red-emitting dye compound, sulforhodamine 101 (**SR 101**) as an organic guest molecule. The high photoluminescence quantum yield of **SR 101** is not typical of many red-emitting systems; indeed, the previously discussed red-emitting phosphorescent dopants **35** (Φ_PL_ = 3 % in MeCN) and **44** (Φ_PL_ = 20 % in MeCN) show much lower quantum yields in solution than **SR 101**. The LEEC produces white light with CIE coordinates coinciding with the white point (CIE 0.33, 0.33). In addition, although the value is still short (*t*_1/2_ = 2.83 h) the device lifetime reported for this device is the longest for white LEECs reported to date [55] However, this is probably due at least in part to the very low luminance levels reported for this device (*L*_max_ = 7.2 cd m^−2^), particularly compared with the devices shown in entries 1 and 2 (Fig. 27).

**Fig. 28:**
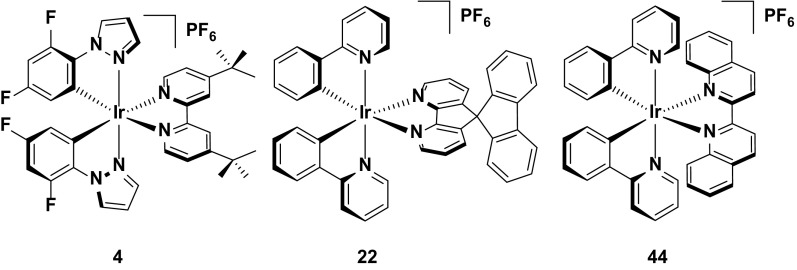
Double-doped host–guest LEEC containing **4** as the blue/green host component, **22** as an orange-emitting electron transporting material and **44** as the red dopant. Device: ITO/PEDOT:PSS/**4** (79.85 wt %), **22** (0.05 wt %), **44** (0.10 wt %), [BMIM][PF_6_] (20.00 wt %)/Al, entry 5

Taking the doping strategy further, Su and co-workers[38c] have demonstrated that these LEECs can be ‘double-doped’, with one dopant providing the red-light component required for white light and the other dopant acting to improve charge transport in the emissive layer, as demonstrated by entry 5. In this case, complex **22** is doped into the emissive layer along with **44**. As mentioned, complex **22** is an effective electron transporting material, acting to improve carrier mobilities throughout the emissive layer and thus improve the efficiency. Indeed, the efficiency of the device (EQE = 6.3 %), entry 5, is almost double the efficiency of the control device utilizing only **4** and **44** in the emissive layer (EQE = 3.2 %). Furthermore, since the orange emission of **22** in the solid state (*λ*_PL_ = 593 nm) falls in between the emission of the host complex **4** (*λ*_PL_ = 492 nm) and the red-emitting dopant **44** (*λ*_PL_ = 672 nm), the overall color of the device is also not negatively impacted (CIE 0.32, 0.43) compared with the control device utilizing only **4** and **44** (CIE: 0.33, 0.42) [56] (Fig. 28).

### Multilayer LEECs

Multilayer LEECs comprise much more varied device architectures than host–guest LEECs. For example, entry 6 is an example where a typical blue-green LEEC employing **4** as the emitter is capped on top of the ITO anode with a color conversion layer (CCL). This CCL is comprised of a transparent inert film doped with a small quantity red dye, such as **DCJTB**, and produces red light following photoexcitation by the blue-emitting component of the LEEC. Control of the doping concentration controls the relative contribution of blue and red light, and thus enables the generation of white light. Furthermore, this device achieves much higher efficiencies (EQE = 12.5 %) than the values reported for the host–guest LEECs in entries 1–5.

**Fig. 29:**
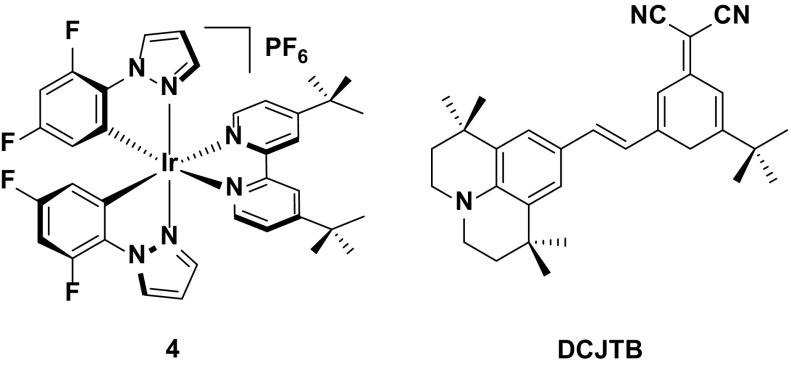
Multilayer LEEC employing **4** as the blue/green component and DCJTB as the red-emitting dopant in the color conversion layer. Device: glass/photoresist film (99.7 wt %), DCJTB (0.3 wt %)/ITO/PEDOT:PSS/**4** (80.0 wt %), [BMIM][PF_6_] (20.0 wt %)/Ag, entry 6

However, an issue with this class of device is that since the chromophores generating white light are not excited simultaneously—as in the case of a typical host–guest device—the color of the device is strongly dependent on the amount of blue light output from the device. Since LEECs characteristically show variation in the luminance as a function of time, this ultimately leads to strong fluctuations in the CIE coordinates of the device with time. The degree of variability is dependent on the concentration of dye present in the CCL, with 0.3 wt% showing the least change in CIE coordinates, but nevertheless the coordinates are not consistent over a 0.5 h period [45, 54] (Fig. 29).

**Fig. 30:**
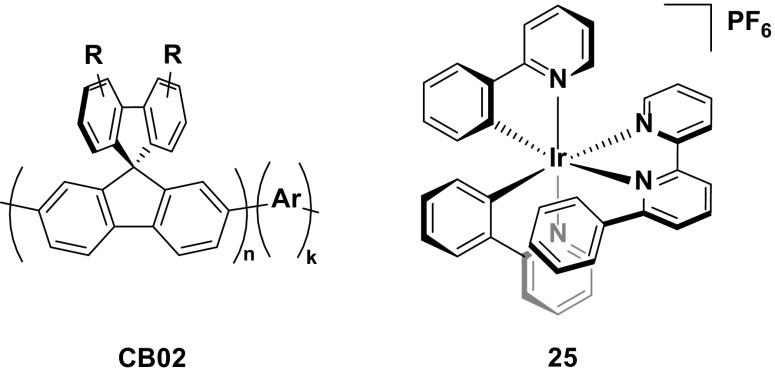
Multilayer LEEC employing **25** as the orange-emitting layer and CB02 as the blue-emitting layer. Device: ITO/PEDOT:PSS/**25**, [BMIM][PF_6_]/CB02/Ba/Ag, entry 7

Since the performance of many white LEECs is ultimately governed by the performance of the blue-emitting iTMC, the device shown in entry 7 addresses this issue by doing away with a blue-emitting iTMC altogether, instead adopting a conjugated polyfluorene polymer **CB02** (the full structure is not disclosed, appearing in the report exactly as shown in Fig. 30) as the blue-emitting component. The device conveniently takes advantage of the ‘orthogonal’ solubility profiles of the iTMC **25** (spin-coated from MeCN) and **CB02** (spin-coated from mesitylene), meaning that each layer can be sequentially deposited from solution without impacting the integrity of previously deposited layer, before encapsulation within the electrodes.

In contrast to the device in entry 6, both emitters are excited electrically, with electrons injected into the **CB02** layer via the cathode and holes into the iTMC layer via the anode, recombining towards the interface of the two emissive layers. Since the **CB02** layer is not charged, this device does not display typical ‘LEEC behavior’, showing no *J*-*V* dependence as a function of time. This probably also accounts for the somewhat high driving voltages utilised for this device (7.0–10.0 V) compared with those discussed previously. Such high voltages also account for the much higher brightness for this device (*L*_max_ = ca. 400 cd m^−2^ at 7.0 V) compared with other white LEECs but this also appears to impact the efficiency, with only a relatively low current efficiency reported (CE = 0.41 cd A^−1^). Nevertheless, by controlling the relative thickness of the emissive layers (and thus the amount of light generated from each emitter), almost ideal white light can be achieved for this device (CIE 0.32, 0.34) [79].

**Fig. 31:**
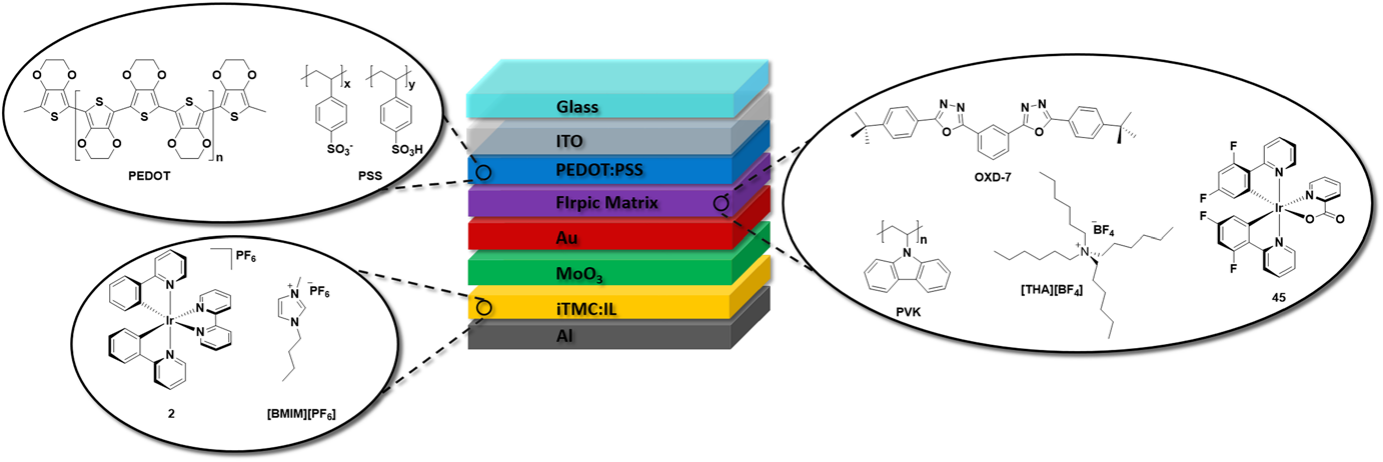
Multilayer LEEC employing **2** as the yellow emitting layer and **45** as the blue-emitting layer. Device: ITO/PEDOT:PSS/PVK (43.5 wt %), OXD-7 (43.5 wt %), **45** (8.7 wt %), [THA][BF_4_] (4.3 wt %)/Au/MoO_3_/**2** (80.0 mol %), [BMIM][PF_6_] (20.0 mol %)/Al, entry 8, where PVK is poly(vinyl-carbazole), OXD-7 is 1,3-bis(5-(4-(*tert*-butyl)phenyl)-1,3,4-oxadiazol-2-yl)benzene and [THA][BF_4_] is tetrahexylammonium tetrafluoroborate

Arguably the most complex device is that of entry 8, which adopts a ‘tandem LEEC’ architecture to achieve white light. In this architecture, two sub-devices emitting yellow (**2**) and blue (‘FIrpic’, complex **45**) light are stacked on top of each other, with an air stable middle electrode employed to separate the two devices. As with entry 7, entry 8 borrows from well-established OLED literature to circumvent the issues with blue-emitting iTMCs, utilizing instead the well-known sky-blue-emitting FIrpic complex. The blue LEEC adopts a typical OLED-type emissive layer, with the neutral complex **45** doped into a host of poly(*N*-vinylcarbazole) (PVK), which has hole transporting characteristics, and 2,2′-(1,3-phenylene)bis[5-(4-*tert*-butylphenyl)-1,3,4-oxadiazole] (OXD-7, Fig. 31), which is an electron transporting material. An ionic liquid dopant (tetrahexylammonium tetrafluoroborate, [THA][BF_4_]) confers characteristic LEEC-type behavior, with the control device, ITO/PEDOT:PSS/PVK (43.5 wt %), OXD-7 (43.5 wt %), **45** (8.7 wt %), [THA][BF_4_] (4.3 wt %)/Au, showing strong variations in luminance as a function of time. The use of a semi-transparent Au cathode allows for deposition of the second yellow-emitting component. In order to preserve the integrity of the Au surface, a thin MoO_3_ interlayer is also deposited. The second emissive layer of **2** is then added, followed by capping the device with an Al cathode.

Whereas entry 7 does not show normal LEEC behavior, the tandem LEEC, like the control based on **45**, does show features typical of LEEC devices: upon applying a constant current, the driving voltage of the device drops rapidly, as ions in the emissive layers migrate to form the dynamically doped zones that facilitate the injection of electrons and holes. This phenomenon is common for conventional LEECs, but this is the only example of it being demonstrated in a double-stacked ‘tandem LEEC’ based on iridium. Overall, the performance of the device is very high: the luminance levels reported for this device (*L*_max_ = 845 cd m^−2^) are by far the highest reported for a white LEEC, while the efficiency (CE = 8.5 cd A^−1^) is comparable to some of the best host–guest LEECs described above. However, the CIE coordinates are somewhat shifted from the ideal white coordinate to ‘warm white’ (CIE 0.38, 0.47).

## Conclusions and Outlook

LEEC research is burgeoning owing to their attractively simple device architectures and facile processing from solution. These two features make these devices appealing for industrial applications, overcoming some of the fabrication drawbacks linked to OLEDs, which rely on costly multilayer device architectures that are typically fabricated by vacuum deposition methods.

In spite of the intrinsic simplicity of LEECs, recent developments in areas from the device architectures (host–guest systems, charge transport layers), to the synthesis of iTMCs (particularly silver-assisted methods) and methods of device operation (pulsed current LEECs) have culminated in reports demonstrating high device efficiencies (EQEs >10 %) and stabilities (*t*_1/2_ of thousands of hours). However, even with these advances, the best performing LEECs fall well short of their OLED counterparts, with stabilities in LEECs in particular at least an order of magnitude lower.

Thus, the principal goal for researchers is still to address issues with respect to LEEC stability. In particular, LEECs still perform particularly poorly in the blue, deep-red, and white. There is therefore a dire need of further advances if these devices are to approach the performance metrics of leading OLEDs. In addition to more established strategies such as incorporating hydrophobic units within the ligand scaffold, several different approaches have begun to emerge that may tackle the difficulties present with devices of these colors. These include polymer-iTMC LEECs to the use of neutral blue emitters to overcome the lack of stable and efficient blue iTMCs. If the challenges relating to LEEC stability and the development of deep-blue devices can be overcome then the future of LEECs will be bright indeed.
